# Predictive Factors for Sustained Virological Response after Treatment with Pegylated Interferon α-2a and Ribavirin in Patients Infected with HCV Genotypes 2 and 3

**DOI:** 10.1371/journal.pone.0107592

**Published:** 2014-09-19

**Authors:** Claus Niederau, Stefan Mauss, Andreas Schober, Albrecht Stoehr, Tim Zimmermann, Michael Waizmann, Gero Moog, Stefan Pape, Bernd Weber, Konrad Isernhagen, Petra Sandow, Bernd Bokemeyer, Ulrich Alshuth, Hermann Steffens, Dietrich Hüppe

**Affiliations:** 1 Clinic for Internal Medicine, St. Josef Hospital, Oberhausen, Germany; 2 Center for HIV and Hepatogastroenterology, Duesseldorf, Germany; 3 Center of Gastroenterology, Goettingen, Germany; 4 ifi-Institute for Interdisciplinary Medicine, Hamburg, Germany; 5 1st Medical Clinic, Dept. Gastroenterology and Hepatology, Johannes Gutenberg University of Mainz, Mainz, Germany; 6 Medcenter Leipzig, Leipzig, Germany; 7 Center of Gastroenterology, Kassel, Germany; 8 Center of Gastroenterology, Paderborn, Germany; 9 Competence Center for Addiction, Praxiszentrum Friedrichsplatz, Kassel, Germany; 10 Practice for general medicine, Koeln, Germany; 11 General Practice, Berlin, Germany; 12 Gastroenterology Practice, Minden, Germany; 13 Virology, Roche Pharma AG, Grenzach-Wyhlen, Germany; 14 Practice for internal Medicine, Berlin, Germany; 15 Center of Gastroenterology, Herne, Germany; National Institutes of Health, United States of America

## Abstract

**Background:**

Previous trials have often defined genotype 2 and 3 patients as an “easy to treat” group and guidelines recommend similar management.

**Aims:**

The present study looks for differences between the two genotypes and analyzes predictive factors for SVR.

**Methods:**

Prospective, community-based cohort study involving 421 physicians throughout Germany. The analysis includes 2,347 patients with untreated chronic HCV genotype 2 (n = 391) and 3 (n = 1,956) infection treated with PEG-IFN α-2a plus ribavirin between August 2007 and July 2012.

**Results:**

When compared with genotype 2 patients, those with genotype 3 were younger, had a shorter duration of infection, lower values of total cholesterol, LDL cholesterol and BMI, a higher frequency of drug use as infection mode and male gender (p<0.0001, respectively), and a higher APRI score (p<0.005). SVR was higher in genotype 2 when compared with genotype 3 (64.7% vs. 56.9%, p = 0.004). By multivariate analysis of genotype 2 patients, low baseline γ -GT and RVR predicted SVR. In genotype 3 age ≤45 years, cholesterol>130 mg/dl, a low APRI score, and a γ-GT ≥3-times ULN, RVR, and RBV starting dose were associated with SVR by multivariate analysis.

**Conclusions:**

The present study corroborates that liver fibrosis is more pronounced in genotype 3 vs. 2. SVR is higher in genotype 2 versus genotype 3 partly because of follow-up problems in genotype 3 patients, in particular in those infected by drug use. Thus, subgroups of genotype 3 patients have adherence problems and need special attention also because they often have significant liver fibrosis.

**Trial Registration:**

Verband Forschender Arzneimittelhersteller e.V., Berlin, Germany ML21645 ClinicalTrials.gov NCT02106156

## Introduction

Previous trials combined genotype 2 (GT2) and GT3 patients as an “easy to treat” group and guidelines recommend similar treatment for both genotypes [1]–[11]. Recently it has been suggested that the two genotypes differ and need more specific management [12]–[16]. Even after the approval of sofosbuvir by FDA and EMA the combination therapy with pegylated interferon and ribavirin will stay an important treatment option for patients infected with GT2 and GT3 in many parts of the world. The present study looks for differences between these genotypes and analyzes predictive factors for sustained virological response (SVR) after treatment with pegylated interferon and ribavirin under real-life conditions.

## Methods

The study was approved by health authorities and the ethical committee Ethik-Kommission der Ärztekammer Westfalen-Lippe, Münster, Germany. This study and all related studies for this drug are registered in the national register for non-interventional studies at vfa (Verband Forschender Arzneimittelhersteller e.V., Berlin, Germany). Written informed consent was obtained from all patients. The protocol for this trial and the supporting TREND checklist are available as supporting information; see Checklist S1 and Protocol S1.

### Patients

In the ongoing observational study ML21645, 9,679 patients with chronic hepatitis C were treated with PEG-IFN α2a and ribavirin (Pegasys, Roche Pharma AG, Grenzach-Wyhlen, Germany in combination with different, in Germany approved and available ribavirins) between August 2007 and July 2012. The present analysis includes all 2,347 patients with untreated GT2 (n = 391) and GT3 (n = 1,956) infection who had a follow-up of at least 24 weeks after end of the antiviral therapy by July 2012 in order to assess SVR24. GT1 data of this cohort have been published previously [17]–[19]. Throughout Germany, 421 physicians (360 in private practice, 61 in hospital settings) contributed a mean of 17 patients. Exclusion criteria were age <18 years and presence of Child B/C cirrhosis. A CONSORT flowchart is provided as Figure S1.

### Definitions of response

Rapid virological response (RVR) was defined as a negative HCV-RNA (<50 IU/ml) 4 weeks after begin of therapy (measured between days 25–30 after treatment start). Early Virological response (EVR) was defined as a ≥2 log_10_ decline from baseline in HCV-RNA or as a negative HCV-RNA (<50 IU/ml) at week 12 (measured between days 74–94). SVR was defined as negative HCV-RNA 24 weeks after end of treatment. The response definitions were the ones used in German and US guidelines [1], [3], [20]. We are aware that in the recent EASL guideline [2] EVR is defined differently as a HCV-RNA detectable at week 4, but undetectable at week 12.

### Statistics

Fisher's exact χ^2^-tests without correcting for multiple testing were used to investigate differences between baseline characteristics and the association between various early response categories (at week 4 or week 12) and SVR, respectively. All statistical tests were exploratory and performed at a two-sided significance level of 0.05.

For continuous variables, receiver operating characteristic (ROC) analyses estimated the best cut-off point for prediction of SVR; these were 45 years for age, 400,000 IU/mL for baseline HCV-RNA, 130 mg/dl for total cholesterol, 85 mg/dl for LDL cholesterol, and 3-times elevation of γ-GT above the upper limit of normal (ULN). Categorical variables were used for continuous variables using these cut-off points. Associations with SVR were analyzed by uni- and multivariate means. Liver biopsy or elastography data were available in only a few patients. Thus, the degree of fibrosis was estimated by APRI score [21] using three categories: a) <0.5; b) 0.5–1.5; and c)>1.5. Values <0.5 exclude significant fibrosis, values between 0.5–1.5 are associated with mild fibrosis and values>1.5 with severe fibrosis or cirrhosis [21]–[24]. For duration of therapy three categories were used: a) <22 weeks, b) 22–26 weeks, and c)>26 weeks [1]–[3].

## Results

### Differences in characteristics between GT2 and GT3 patients

GT2 patients were on average 7.1 years older than GT3 patients (p<0.0001); correspondingly, the estimated duration of infection was 3.8 years longer in GT2 vs. GT3 patients (p<0.0001). In GT3 patients 69.3% were male whereas in GT2 only 58.6% were male (p<0.0001). Among GT3 patients drug use accounted for 61.9*%* of infections whereas this infection mode was seen in only 36.8% of GT2 patients (p<0.0001). Prior administration of blood products accounted for 12.3% of GT2 infections but only for 4.8% of GT3 infections (p<0.0001) (Table 1).

**Table 1:** Main characteristics of patients infected with GT2 and GT3.

	GT2		GT3		p
	%	n/total	%	n/total	
Male gender	58.6%	229/391	69.3%	601/1956	<0.0001
HIV co-infection	2.6%	10/391	3.2%	62/1956	0.631
HBV co-infection	2.3%	9/391	2.2%	43/1956	0.851
Current drug use	21.5%	84/391	42.6%	834/1956	<0.0001
Drug substitution	10.5%	41/391	24.8%	486/1956	<0.0001
Alcohol abuse #	2.3%	9/391	4.8%	93/1956	0.029
Psychiatric co-morbidity	14.6%	57/391	16.0%	312/1956	0.534
Depression	10.2%	40/391	12.5%	245/1956	0.235
Psychosis	3.1%	12/391	2.5%	49/1956	0.489
Psychoanaleptics	13.3%	52/391	19.9%	390/1956	0.007
Cardiovascular disease	10.0%	39/391	4.6%	89/1956	<0.0001
Beta-blocking agents	5.4%	21/391	2.6%	50/1956	0.005
Calcium channel blockers	2.3%	9/391	0.6%	11/1956	0.003
Agents acting on renin-angiotensin	5.4%	21/391	2.4%	47/1956	0.003
Diabetes mellitus	5.9%	23/391	1.1%	52/1956	0.002
Anti-diabetic agents	4.1%	16/391	1.2%	23/1956	<0.0001
*Modes of infection*					
Blood products	12.3%	48/391	4.8%	94/1956	<0.000
Drug use (i.v. or nasal)	36.8%	144/391	61.9%	1211/1956	<0.000
Sexual transmission	4.3%	17/391	5.0%	97/1956	0.700
Tatoos/piercing	1.8%	7/391	2.5%	48/1956	0.582
Needle stick	1.5%	6/391	1.4%	27/1956	0.814
Surgical/medical procedure	5.9%	23/301	2.4%	46/1956	0.001
Medical/dental profession	1.8%	7/391	0.7%	14/1956	0.068
Unknown	37.9%	148/391	24.3%	476/1956	<0.000
SVR	64.7%	253/391	56.9%	1112/1956	0.004
RVR *	68.2%	131/192	68.0%	613/902	1.000
EVR **	93.0%	254/273	92.6%	1133/1223	0.898
SVR in RVR patients	73.3%	96/131	65.9%	404/613	0.124
SVR in EVR patients	72.8%	185/254	66.6%	755/1133	0.056
Baseline HCV-RNA > 400.000 IU/ml	58.7%	229/390	50.8%	983/1935	0.004

# alcohol abuse was assessed by judgement of the physician.

* only patients who were treated at least for 4 weeks and in whom RVR was correctly determined (see Methods for further details).

** only patients who were treated at least for 12 weeks and in whom EVR was correctly determined (see Methods for further details).

The rate of patients who recently used drugs was higher in GT3 versus GT2 (42.6 vs. 21.5%; p<0.0001) (Table 1); similarly drug use as an infection mode was also more frequent in GT3 (Table 1). On the other hand the infection modes of blood products, medical procedures and those of unknown etiology were more frequent in GT2 patients when compared with GT3 (Table 1). Although GT2 patients were older, had a longer disease duration and a higher BMI when compared with GT3, the APRI score was higher in GT3 vs. GT2 (1.0 v. 0.8; p = 0.005). The rates of HIV- and HBV-coinfections were similar in the two genotypes as well as the rate of psychiatric comorbidity including depression and psychosis (Table 1). However, GT3 patients had a higher rate of drug substitution and a higher rate of taking psychoanaleptic drugs (Table 1). The rates of most other co-morbidities and co-medications were similar in the two genotypes (data without significant differences not shown) except for cardiovascular disease and diabetes mellitus which were more frequent in GT2 versus GT3. Correspondingly, GT2 patients took antihypertensive agents like beta-blockers, calcium channel blockers, and agents acting on the renin-angiotensin system more frequently (Table 1).

The rate of alcohol abuse was relatively low in both genotypes (<5%), but higher in patients infected with GT2 versus those infected with GT3 (Table 1). The APRI score was higher in patients with alcohol abuse (1.3±1.2 vs. 1.0±1.3, p = 0.025); this difference in the APRI score was seen tendentially both in GT2 (APRI score with versus without alcohol abuse 1.3±1.8 and 0.8±1.1, p = 0.418), respectively) and in GT3 (APRI score with versus without alcohol abuse 1.3±1.2 and 1.0±1.3, p = 0.070) respectively).

Baseline values for platelets, prothrombine time, ferritin, and γ-GT were not significantly different between genotypes, although platelets and prothrombine time tended to be lower and γ-GT tended to be higher in GT3 when compared with GT2 (Table 2).

**Table 2:** Main characteristics of patients infected with GT2 and GT3 (Mean).

	mean	SD	n	mean	SD	n	
Age (years)	45.4	12.1	391	38.3	9.6	1956	<0.0001
Duration of infection (years)	13.9	10.2	332	10.1	7.8	1743	<0.0001
BMI	25.9	4.7	391	24.9	4.1	1956	<0.0001
APRI score	0.8	1.1	346	1.0	1.3	1780	0.005
Thrombocytes/nl	224	75	376	220	72	1885	0.400
Prothrombine time (%)	97.5	12.0	245	96.9	14.2	1155	0.124
γ-GT (U/L)	68	109	370	79	104	1872	0.061
Cholesterol (mg/dl)	194	40	220	166	53	1037	<0.0001
LDL cholesterol (mg/dl)	116	34	120	95	36	532	<0.0001
Triglycerides (mg/dl)	130	92	210	119	77	945	0.073
Ferritin (µg/L)	193	410	150	165	173	657	0.409
Duration of therapy (weeks)	24.8	8.8	391	24.4	10.0	1950	0.492
RBV starting dose (mg/kg/day)	12.6	3.0	390	12.6	3.4	1950	0.767

The mean body mass index (BMI) was higher for GT2 vs. GT3 (25.9 vs. 24.9; p<0.0001) (Table 2). This difference is not explained by an increase in BMI with older age because this correlation was not statistically significant (data not shown). BMI was similar for both gender in GT2 but higher for males in GT3 (Table 3). Baseline cholesterol was similar for both genders in GT3 but higher for females in GT2. LDL cholesterol was similar for both genders (data not shown). Both total and LDL cholesterol were higher in GT2 when compared with GT3 (194 vs. 166 mg/dl, p<0.0001; 116 vs. 95 mg/dl, p<0.001) (Table 2).

**Table 3:** BMI and baseline cholesterol versus gender.

Body Mass Index (kg/m^2^)			
Genotype 2			p = 0.760
	mean	SD	N
Male	25.9	4.0	229
Female	26.0	5.5	162
Total	25.9	4.7	391
Genotype 3			p<0.0001
	mean	SD	N
Male	25.2	3.8	1355
Female	24.3	4.6	601
Total	24.9	4.1	1956
Baseline Cholesterol (mg/dl)			
Genotype 2			p = 0.011
	mean	SD	N
Male	188.4	40.7	139
Female	202.6	37.8	81
Total	193.6	40.2	220
Genotype 3			p = 0.171
	mean	SD	N
Male	164.4	57.3	727
Female	169.3	40.7	310
Total	165.9	52.9	1037

Differences in BMI, total cholesterol and LDL cholesterol between patients infected with different genotypes are not explained by differences in age and gender because these correlations were not statistically significant (data not shown). Triglyceride levels were not different for GT2 vs. GT3 although the levels were slightly higher in GT2 (130 vs. 119 mg/dl; P = 0.073).

### SVR, RVR, and EVR

SVR was higher in GT2 when compared with GT3 (64.7 vs. 56.9%, p = 0.004) (Table 1). In GT3 patients SVR was 66.6% in those with an EVR and 21.1% in those without. In GT3 patients without an EVR, therapy was discontinued in week 12 in only 13/90 patients with a SVR of 0/13. Despite the non-response at week 12 and the corresponding stop rule, therapy was continued in 77/90 patients with a SVR of 24.7% (19/77). Of these 19 non-EVR patients with SVR, ten (53%) were treated>24 weeks (range: 26–52 weeks). In GT2 patients SVR was 72.8% in those with an EVR and 26.3% in those without. In 19 GT2 patients without an EVR therapy was discontinued in week 12 in only five (26.3%) with SVR of 0%. Despite this non-response therapy was continued in 14 of 19 patients resulting in a SVR of 35.7% (5/14). Of five non-EVR patients with SVR four (80%) were treated>24 weeks (range: 26–52 weeks).

For further EVR analyses we excluded patients who discontinued treatment prior to week 12 as well as those in whom viral load was measured prior to day 74 or later than day 94 after start of treatment. EVR was similar for GT2 and GT3 (93.0 vs. 92.6%, p = 0.9). When assuming similar conditions as explained for EVR, RVR was similar for GT2 vs. GT3 (68.2 vs. 68.0%; p = 1.0).

We also determined in how many patients EVR was measured according to the German and EASL guidelines; for this analysis we excluded patients who discontinued treatment prior to week 12. It was also assumed that EVR was not correctly assessed when HCV-RNA was measured prior to day 74 or later than day 94. EVR was correctly determined in 273/372 patients (73.4%) for GT2 and in 1,223/1,894 patients (64.6%) for GT3. When assuming similar conditions as explained for EVR, RVR was correctly determined in 902/1,895 GT3 patients (47,6%) and in 192/388 GT2 patients (49.5%). Detailed data on the number of patients in whom EVR and RVR were determined correctly, i.e. as suggested by the corresponding guidelines, are given in Table 1.

SVR in patients with EVR or RVR was also determined by excluding the type of patients explained above. For GT3 SVR was 65.9% in patients with RVR and 48.4% in patients without. For GT2 SVR was 73.3% in patients with RVR and 44.3% in patients without.

Total duration of treatment and initial RBV doses were similar for both genotypes (Table 2). Subtypes of genotypes were associated with SVR in neither GT2 nor GT3 (data not shown).

When SVR was calculated only for patients with a planned end of therapy or with a discontinuation because of virological failure or side-effects, SVR was 70.5% for GT2 (253/359) and 67.6% for GT3 (1,112/1,645) (p = 0.317); when these results are compared with SVR in the total group, SVR is increased by 10.7% in GT3 but only by 5.8% in GT2 when applying this type of analysis. We therefore looked for genotype-specific differences in the follow-up of patients with an end-of-treatment (EOT) response. Of the total 1,454 GT3 patients with an EOT response, SVR was not determined in 209 patients (14.4%), in particular because patients were lost to follow-up. In contrast, SVR was not determined for such reason in only 27 of 315 GT2 patients (8.6%).

Further analyses looked for explanations for these genotype-specific differences. One analysis looked at the correlation between genotype and mode of infection, revealing that patients with an infection mode of drug use had a lower SVR than those with other infection modes for GT3 but not for GT2 (Table 4). In addition, we analyzed the loss to follow-up in EOT responders in relation to their infection mode. The highest loss to follow-up was seen in patients with drug use as the infection mode with 12.5 and 15.1% in GT2 and GT3, respectively; these percentages were higher when compared to patients with other infection modes (4.4 and 8.2% for GT2 and GT3; p = 0.008 and p<0.0001, respectively).

**Table 4:** SVR versus mode of infection in GT2 and GT3 patients.

	Genotype 2					Genotype 3			
	No SVR		SVR		p	No SVR		SVR	
	N	%	N	%		N	%	N	%
Blood products	15	31.3	33	68.8	0.629	35	37.2	59	62.8
Drug use (i.v. or nasal)	58	40.3	86	59.7	0.100	550	45.4	661	54.6
Sexual transmission	7	41.2	10	58.8	0.609	42	43.3	55	56.7
Tatoos/piercing	3	42.9	4	57.1	0.700	21	43.8	27	56.3
Needle stick	3	50.0	3	50.0	0.427	11	40.7	16	59.3
Surgical/medical procedure	9	39.1	14	60.9	0.659	13	28.3	33	71.7
Medical/dental profession	1	14.3	6	85.7	0.429	4	28.6	10	71.4
Unknown	46	31.1	102	68.9	0.227	187	39.3	289	60.7

(multiple answers allowed; differences between modes of infections in GT2 versus GT3 were tested for significance by Fisher's exact χ^2^-test without correcting for multiple testing).

Because of the low rate of determining EVR, we looked whether this phenomenon might be due to the fact that physicians did not determine HCV-RNA at week 12 when an RVR had been documented in week 4. Interestingly, in GT3 EVR was correctly determined in 666/856 patients in whom RVR was measured (77.8%), while EVR was not determined in 228/401 patients (56.9%) in whom RVR was not determined. This phenomenon was similar for GT2 (data not shown).

Four out of five GT2 EVR non-responders with a SVR were treated>26 weeks; two of them had a SVR when treated with>800 mg RBV. Ten of 19 GT3 EVR non-responders with a SVR were treated>26 weeks (52.6%) and ten of 19 GT3 EVR non-responders received a RBV dose>800 mg (52.6%).

In this non-interventional study IL28 genotype was determined rarely, in 72/1,956 GT3 patients (3.7%) (CC 70.8, CT 22.2, TT 6.9%) and in 14/391 GT2 patients (3.6%) (CC 71.4, CT 21.4, TT 7.1%). In GT 2 SVR was 60% (6/10) in IL28 CC and 75% (3/4) in IL28 non-CC (p = 0.868); in GT3 SVR was 72.5% (51/67) in IL28 CC and 47.6% (10/21) in IL28 non-CC (p = 0.051).

### Variables predicting SVR

By univariate analysis of GT2 patients, at baseline a high prothrombine time, a low APRI score, and a γ-GT ≤3-times the ULN predicted SVR; RVR and RBV starting dose were also associated with SVR (Table 5). By multivariate analysis only a low γ-GT and RVR were associated with SVR (Table 6).

**Table 5:** Univariate analysis of variables for prediction of SVR in GT2 and GT3.

	Genotype 2					Genotype 3				
Baseline variables	p	OR	95% CI for OR *		N	p	OR	95% CI for OR *		N
Gender	0.805				391	0.156				1956
Age ≤45 vs.>45 years	0.842				391	0.014	0.781	0.642	0.950	1956
BMI	0.843				391	0.364				1956
Subtype of HCV genotype	0.381				224	0.753				1332
Duration of infection (years)	0.910				332	0.800				1743
HCV-RNA ≤400,000 vs.>400,000 IU/ml	0.458				390	0.045	0.832	0.695	0.996	1935
Psychiatric co-morbidity	0.972				391	0.022	0.754	0.591	0.960	1956
HIV co-infection	0.752				391	0.558				1956
HBV co-infection	0.406				391	0.166				1956
Current drug use	0.388				391	0.063				1956
LDL cholesterol ≤85 vs.>85 mg/dl	0.308				120	0.000	2.073	1.456	2.951	532
Cholesterol ≤130 vs.>130 mg/dl	0.332				220	0.000	1.838	1.360	2.483	1037
Triglycerides (mg/dl)	0.962				210	0.564				945
Serum ferritin (µg/l)	0.075				150	0.206				657
Prothrombine time (%)	0.007	1.032	1.009	1.055	245	0.138				1155
APRI score <0.5; 0.5-≤1.5; 1.5-≤2 #	0.050	0.783	0.613	1.000	346	0.000	0.764	0.689	0.846	1780
γ-GT> vs. ≤3-times ULN	0.017	0.392	0.182	0.844	370	0.000	0.480	0.347	0.663	1872
**Variables under treatment**										
RVR	0.000	3.759	2.004	7.052	195	0.000	2.900	2.210	3.803	963
Duration of therapy <22 weeks - 22–26 weeks ->26 weeks	0.247				359	0.732				1643**
Change in dose PEG-interferon	0.326				391	0.140				1953
RBV starting dose (mg/kg body weight)	0.002	1.127	1.046	1.213	390	0.004	1.041	1.013	1.069	1950

* OR  =  Odds Ratio; CI  =  Confidence interval.

** With a planned treatment end or with treatment discontinuation for virological failure or adverse events.

# By univariate analysis baseline thrombocytes and GOT were also significant in GT3 in predicting SVR; in GT2 these variables were not significant. Since the APRI score which combines these two variables had a higher predictive value when compared with the two single variables only the APRI score was used for further analyses.

**Table 6:** Multivariate analysis of variables for prediction of SVR in GT2 and GT3.

	Genotype 2				Genotype 3			
Baseline variables in patients with complete data available *	p	OR	95% CI for OR §		p	OR	95% CI for OR §	
Age ≤45 years vs.>45 years					0.001	0.613	0.460	0.817
Cholesterol ≤130 vs.>130 mg/dl					0.001	1.768	1.277	2.446
γ-GT (U/l)> vs. ≤3 time ULN	0.011	0.341	0.148	0.785	0.030	0.585	0.361	0.950
APRI score <0.5. 0.5 - ≤1.5. 1.5 - ≤2	0.269				0.002	0.786	0.673	0.917
HCV-RNA ≤400,000 vs.>400,000 IU/ml					0.780			
Psychiatric co-morbidity					0.165			
**Variables under treatment in patients with complete data** *								
RVR	0.000	3.770	1.930	7.362	0.001	1.63	1.212	2.203
Ribavirin starting dose (mg/kg body weight)	0.256				0.006	1.069	1.208	2.198

* For GT3 complete baseline data for multivariate analysis were available for 968 patients and in 341 patients with GT2; complete data under treatment were available for 787 patients with GT3 and for 180 patients with GT2.

§ OR  =  Odds Ratio; CI  =  Confidence interval.

In GT3 univariate analysis showed that several baseline variables were associated with SVR: age ≤45 years, total cholesterol>130 mg/dl, LDL cholesterol>85 mg/dl, HCV-RNA ≤400,000 IU/mL, low APRI score, γ-GT ≤3-times ULN and absence of psychiatric co-morbidity (Table 5). By multivariate analysis age ≤45 years, total cholesterol>130 mg/dl, low APRI score and γ-GT ≥3-times ULN were associated with SVR but not HCV-RNA and psychiatric co-morbidity (Table 6). RVR and RBV starting dose were associated with SVR by uni- and multivariate analysis in GT3 (Table 6).

We also did a GT3 multivariate analysis including both total and LDL cholesterol showing that total cholesterol was still significant (p<0.0001) but not LDL cholesterol (p = 0.106) (data not shown). Since LDL was only measured in ¼ of patients, we present multivariate analyses in Table 6 without including LDL data.

## Discussion

Treatment algorithms have been similar for GT2 and GT3 in German, EASL and AASLD guidelines recommending PEG-IFN and RBV at a flat dose of 800 mg/day for 24 weeks [1]–[3]. Studies with DAAs have been successful for GT2, whereas SVR appears to be lower in GT3 [25]–[36]. Recently, sofosbuvir has been approved as the first DAA for patients with GT2 and GT3. Major differences in the response to sofosbuvir-based therapy in GT2 versus GT3 patients remain. These latter data further emphasize that the two genotypes differ in many important aspects [26]. It is unclear in which countries DAAs like sofosbuvir will be reimbursed in view of economic problems [27]. In countries with reimbursement issues therapy with PEG-IFN and RBV will remain the standard. Therefore, it is crucial to optimize this therapy.

It has recently been suggested that GT2 and GT3 differ in various aspects and need more specific management [13]–[16]. In particular, it has been shown that SVR appears to be higher and relapse rate to be lower in GT2 when compared with GT3 [8], [15], [28]. In addition, GT3 has been suggested to cause a more rapid progression towards fibrosis when compared with GT2 [16], [29]–[30]. GT3 induces a specific interaction with cholesterol metabolism and a specific type of hepatic steatosis [31]. GT3 is often associated with a history of drug abuse and adherence problems [32]. There is still a controversy which subgroups of GT2 and GT3 patients need a shortened or prolonged therapy and RBV dosing adapted to body weight [13], [33]–[35]. There is some agreement that treatment shortening should be reserved to patients with RVR and low baseline viral load and that prolongation to those without RVR and with further negative predictive factors [1]–[3], [8], [14]. In previous studies lack of fibrosis predicted SVR, as well as young age and a HCV-RNA <400,000–600,000 IU/ml [5]–[11]. Some studies showed that low BMI [6], [8] was associated with a high SVR. In addition high γ-GT and low platelets appeared to be associated with a low SVR [12], [16]. RVR predicted SVR both in GT2 and GT3 [6], [9], [11]–[12].

The present large real-life study shows that there are many important differences between GT2 and GT3 patients. GT3 patients are younger, have a shorter duration of infection and are more likely to be male than GT2 patients. The predominant mode of infection was drug use in GT3, whereas prior administration of blood products had been documented more often in GT2. The differences in infection modes may partly explain differences in age, gender, and disease duration between patients infected with different genoytpes. Although GT2 patients were older, had a longer disease duration and a higher BMI when compared with GT3, the APRI score and thus the severity of fibrosis was higher in GT3 vs. GT2. This data corroborates that fibrosis progresses more rapidly in GT3 when compared with other genotypes [16], [29]–[30]. Since alcohol abuse was more frequent in patients infected with GT3 versus GT2, alcohol might be a co-factor for the more rapid fibrosis in GT3. However, alcohol abuse is probably not the only explanation for the latter finding because alcohol abuse was seen in less than 5% of patients with either genotype.

In our large real-life cohort SVR in GT2 (64.7%) and GT3 (56.9%) appears relatively low when compared with previous controlled trials [4]–[12]; in line with previous studies SVR was higher in GT2 when compared with GT3. When SVR was calculated only for patients with a planned end of therapy or with a discontinuation because of virological failure or side-effects, SVR was increased by more than 10% in GT3 but only by about 6% in GT2 when compared with the total cohort. Further analyses showed that many GT3 patients with an EOT response were lost to follow-up and thus counted as having no SVR, whereas this phenomenon was less pronounced in GT2. Additional calculations revealed that GT3 patients with an infection mode of drug use had a lower SVR and a higher loss to follow-up than those with other modes of infection. Thus, in particular the relatively low SVR in GT3 is partly explained by loss of follow-up after EOT in patients with a drug history. This data confirms that some GT3 patients have adherence problems [32]. Such adherence issues may also be important in light of new DAA therapies including sofosbuvir of which one tablet costs more than 700 Euro in most European countries.

We are aware that several studies showed that drug adherence and SVR may be quite good also in patients with drug use and in those with methadone maintenance [36]–[39]. Most of these studies, however, were done in clinical trial settings. Some studies showed that SVR was somewhat lower in patients with methadone maintenance when compared to that expected for controls [36]–[37]. In addition, a controlled study in methadone-substituted patients showed that SVR was markedly better with direct observed therapy versus self-administration of pegylated interferon [39]. Thus, even when considering the latter studies we suggest from the present real-world findings that some GT3 patients with a drug history have adherence problems which may reduce SVR. One reason could be that treatment under real-life conditions includes less experienced physicians without a well integrated internist-addiction medicine [40].

In the present cohort, RVR was determined in only about 70% of patients and EVR in only about 50% for both genotypes. Thus, some physicians tend to care neither for shortening of therapy (by not measuring RVR) nor for the stop rule at week 12 (by not measuring EVR). The physicians who do not care for shortening and those who do not care for stop rules tend to be the same. They for patients 24 weeks regardless of viral kinetics. In patients without an EVR most physicians continued treatment with SVR rates exceeding 20%; these SVR values in EVR non-responders are 10-times higher than those reported in RCTs (literature in [1]–[3]). This data is similar to what we have recently reported in a real-life GT1 cohort [19]. In the GT1 cohort we identified factors explaining the high SVR in EVR non-responders in whom treatment was continued despite stop rules [19]. This was partly due to the fact that reductions in PEG-IFN or RBV doses were not performed despite recommendations which are strictly observed in RCTs. We did not assess the latter association in the present cohort because it has a different focus. Nevertheless, the present analysis again shows that it is insufficient to only base guidelines on RCTs which may not mirror clinical reality. The present real-life data also suggests that therapy>24 weeks and a weight-adapted RBV dose may be helpful for patients with a slow initial virological response.

In this non-interventional study, IL28 genotype was determined rarely, and therefore this data was not included in analyses for prediction of SVR. Nevertheless, the present data is still interesting by showing that measurement of IL28 genotype is not considered important in the broad medical community in Germany for GT2 and GT3 although there is no reimbursement problem for this determination. Since IL28 was measured only in a small fraction of the GT2 and GT3 patients we do not discuss potential differences of IL28 data between the two genotypes. HCV interferes with several pathways of lipid metabolism [31], [41]–[42]. Steatosis is seen in up to half of patients infected with GT3 [43]–[44]; it is directly linked to HCV and often resolves after SVR [45]–[46]. It has recently been shown that total cholesterol but not triglycerides were lower in patients with GT3 when compared to GT2 [47]. Obesity and insulin-resistance are risk factors for steatosis and fibrosis and decrease the chance for SVR in GT1 [43], [45], [48]–[49]. High LDL and cholesterol values as well statin use have been associated with a good SVR in GT1 [47], [50]–[51]. To our knowledge, it has not been reported previously that high values of total and LDL cholesterol are strong predictors for SVR also in GT3, whereas this association is not seen in GT2. In our large cohort we corroborate the findings by Clark et al. [47] showing that both total cholesterol and LDL are markedly lower in GT3 when compared to GT2. The present finding that BMI is higher in patients infected with GT2 versus GT3 was unexpected. We suggest that our results are valid because the statistical difference was large and we also identified that cardiovascular disease, diabetes mellitus, and the use of anti-hypertensive drugs were more frequent in GT2 versus GT3 patients.

In summary, the present study confirms that liver fibrosis is more pronounced in GT3 vs. GT2. SVR is higher in GT2 vs. GT3 partly because of follow-up problems in GT3 patients and in particular in those who had been infected by drug use. Thus, subgroups of GT3 patients have adherence problems and are difficult to treat; these patients may have significant liver fibrosis and need special attention and education as well as intensified treatment.

## Supporting Information

Figure S1 **Study patients.**
(DOCX)

Checklist S1 **TREND Checklist.**
(DOCX)

Protocol S1 **Study protocol ML21645.**
(DOC)
