# Supplementary material for: Predictive Factors for Sustained Virological Response after Treatment with Pegylated Interferon α-2a and Ribavirin in Patients Infected with HCV Genotypes 2 and 3
Source: PLoS One. 2014 Sep 19;9(9):e107592. doi: 10.1371/journal.pone.0107592 (PMC4169557; doi:10.1371/journal.pone.0107592)
Supplement: Figure S1 — Study patients. (DOCX) [file pone.0107592.s001.docx]

**Figure 1. Study patients**
