# Supplementary material for: Predictive Factors for Sustained Virological Response after Treatment with Pegylated Interferon α-2a and Ribavirin in Patients Infected with HCV Genotypes 2 and 3
Source: PLoS One. 2014 Sep 19;9(9):e107592. doi: 10.1371/journal.pone.0107592 (PMC4169557; doi:10.1371/journal.pone.0107592)
Supplement: Protocol S1 — Study protocol ML21645. (DOC) [file pone.0107592.s003.doc]

Noninterventional Study

**Peginterferon alfa-2a (Pegasys®)**

**Noninterventional study for quality assurance in therapy of chronic hepatitis C with Peg-(40kd)-Interferon alfa-2a (Pegasys®) and ribavirin (i.e. Copegus**®**) at hepatology centers – a project in cooperation with BNG (Association of German Resident Gastroenterologists, part III)**

**Observational Protocol**

(ML21645**)**

**Version 4.3 of 28-Feb-2013**

Consolidated version including Amendment 3

**Roche Pharma AG**Grenzach-Wyhlen

TABLE OF CONTENTS

1 Contact [4](#__RefHeading___Toc382480412)

2 List of abbreviations [5](#__RefHeading___Toc382480413)

3 Synopsis [6](#__RefHeading___Toc382480414)

4 Objectives and rationale of the noninterventional study [9](#__RefHeading___Toc382480415)

4.1 Background [9](#__RefHeading___Toc382480416)

4.1.1 Introduction [9](#__RefHeading___Toc382480417)

4.1.2 Quality assurance in medicine [9](#__RefHeading___Toc382480418)

4.1.3 Hepatitis C [10](#__RefHeading___Toc382480419)

4.2 Formulation of the research question [11](#__RefHeading___Toc382480420)

4.3 Rationale for choice of method [13](#__RefHeading___Toc382480421)

5 Selection criteria [13](#__RefHeading___Toc382480422)

5.1 Procedure for selection of participating physicians [13](#__RefHeading___Toc382480423)

5.2 Description of patient access [14](#__RefHeading___Toc382480424)

6 Conduct of the noninterventional study [14](#__RefHeading___Toc382480425)

6.1 Duration and premature termination of the noninterventional study [14](#__RefHeading___Toc382480426)

6.2 Procedure [14](#__RefHeading___Toc382480427)

7 Description of nature and scope of documentation [15](#__RefHeading___Toc382480428)

7.1 General structure of the case record form [15](#__RefHeading___Toc382480429)

7.2 Survey variables [15](#__RefHeading___Toc382480430)

7.2.1 Practice characteristics (multiple responses possible) [15](#__RefHeading___Toc382480431)

7.2.2 Main module for recording treatment of hepatitis C [16](#__RefHeading___Toc382480432)

7.2.3 Additional module for recording treatment of hepatitis C in opioid substituted patients [22](#__RefHeading___Toc382480433)

7.2.4 Additional module for recording therapy of hepatitis C in HIV coinfected patients [24](#__RefHeading___Toc382480434)

7.2.5 Additional module to record treatment of hepatitis C in patients with cardiovascular risk [25](#__RefHeading___Toc382480435)

7.2.6 Additional module to record treatment of hepatitis C in patients after liver transplant [28](#__RefHeading___Toc382480436)

7.2.7 Pharmacoeconomics [30](#__RefHeading___Toc382480437)

7.2.8 Recording of adverse events and pregnancies [33](#__RefHeading___Toc382480438)

7.3 Influencing and interfering variables [34](#__RefHeading___Toc382480439)

7.4 Adverse events [34](#__RefHeading___Toc382480440)

7.5 Expedited reporting procedure [34](#__RefHeading___Toc382480441)

7.6 Pregnancies [35](#__RefHeading___Toc382480442)

8 Statistical aspects [36](#__RefHeading___Toc382480443)

8.1 Number of patients [36](#__RefHeading___Toc382480444)

8.2 Choice of centres [36](#__RefHeading___Toc382480445)

8.3 Analysis plan [36](#__RefHeading___Toc382480446)

9 Report [36](#__RefHeading___Toc382480447)

10 Administrative aspects [37](#__RefHeading___Toc382480448)

10.1 Ethical advice [37](#__RefHeading___Toc382480449)

10.2 Legal principles and registration [37](#__RefHeading___Toc382480450)

10.3 Quality assurance measures [37](#__RefHeading___Toc382480451)

10.3.1 Monitoring [37](#__RefHeading___Toc382480452)

10.3.2 Data management [37](#__RefHeading___Toc382480453)

10.3.3 Archiving [38](#__RefHeading___Toc382480454)

10.4 Public study registry and publication [38](#__RefHeading___Toc382480455)

11 Reimbursement and fees [38](#__RefHeading___Toc382480456)

12 Signatures [40](#__RefHeading___Toc382480457)

13 References [41](#__RefHeading___Toc382480458)

14 Annexes [44](#__RefHeading___Toc382480459)

14.1 Patient information and informed consent [44](#__RefHeading___Toc382480460)

14.2 German Local Drug Safety Bilingual RO-GNE: Adverse Event Form (English/German) [44](#__RefHeading___Toc382480461)

14.3 German Local Drug Safety Bilingual RO-GNE: Pregnancy Record Form (English/German) [44](#__RefHeading___Toc382480462)

# Contact

Serious adverse events and adverse events required to be reported in the expedited procedure are recorded by the physician within 24 h in the AE documentation of the eCRF and sent (automatically) to the Drug Safety Department of Roche Pharma AG (see Section 7.5). Furthermore, a pregnancy must be documented within 24 h and reported by email (or by fax) in English to the Drug Safety Department of Roche Pharma AG using the Pregnancy Report Form (Annex 14.3).

In the event of technical failure of the eCRF, please use the bilingual AE report form "Adverse Event Form" (see Annex 14.2) to report the events in Section 7.5 and send it within 24 h by email (or by fax) in English to the following address:

**Drug Safety Department, Roche Grenzach:**

Address: Roche Pharma AG

Drug Safety Department

Emil-Barell-Straße 1

D-79639 Grenzach-Wyhlen

Telephone No.: +49 7624 14 3707

Fax No.: +49 7624 14 3183

Email: grenzach.drug_safety@roche.com

Please address any questions relating to the procedure of the noninterventional study to:

**CRO Project Leader:** Heike Pfeiffer-Vornkahl

Address: Faktum GmbH

Kaiserstraße 51

D-63065 Offenbach

Telephone No.: +49 69 829714-15

Fax No.: +49 69 8004924

Email: pfeiffer-vornkahl@factum.com

**Medical Manager, Roche Grenzach**

Dr. Ulrich Alshuth

Address: Roche Pharma AG

Emil-Barell-Straße 1

D-79639 Grenzach-Wyhlen

Telephone No.: +49 7624 14 2407

Fax No.: +49 7624 14 3212

Email: ulrich.alshuth@roche.com

**Study Program Manager, Roche Grenzach**

Dr. Heike Berthold

Address: Roche Pharma AG

Emil-Barell-Straße 1

D-79639 Grenzach-Wyhlen

Telephone No.: +49 7624 14 2974

Fax No.: +49 7624 6859

Email: heike.berthold@roche.com

# List of abbreviations

| Abuse | According to Directive 2001/83/EC, Article 1, Paragraph 16 "persistent or sporadic intentional excessive use of medicinal products which is accompanied by harmful physical or psychological effects." |
| --- | --- |
| AE | Adverse Event |
| AMG | German Medicines Act |
| AMS | Drug Safety Department |
| Occupational exposure | This refers to occupational exposure to a medicinal product for human use |
| CRF | Case Record Form |
| DCC | Drug Counselling Centre |
| EoFU | End of Follow-up |
| EOT | End of Treatment |
| EVR | Early Virological Response |
| JFC | Joint Federal Committee |
| HCV | Hepatitis C Virus |
| HIV | Human Immunodeficiency Virus |
| IU | International Units |
| Medication error / unintentional | Medication errors and potential medication errors are all avoidable events which cause / could cause or could lead to unsuitable incorrect use of medications or harm to patients while the medication is under the control of a health care professional, the patient or the consumer. |
| Misuse | Improper use or deliberate misuse is the persistent or sporadic, intentional and excessive use or misuse in a manner not in conformity with the product information or established medical practice or which has no relation to it. |
| NIS | Noninterventional study |
| QMS | Quality Management System |
| RVR | Rapid Viral Response |
| SAE | Serious Adverse Event |
| SVR | Sustained Virological Response |
| Overdose | An overdose is the administration of a dose of a medicinal product (single administration or cumulative dose) which is above the maximum recommended dose approved in the marketing authorization. |

# Synopsis

| AIMS/CTMS No.: | ML21645 – Amendment 2 |
| --- | --- |
| Title: | Noninterventional study for quality assurance in therapy of chronic hepatitis C with Peg-(40kd)-Interferon alfa-2a (Pegasys®) and ribavirin (i.e. Copegus®) at hepatology centers – a project in cooperation with BNG (Association of German resident Gastroenterologists, part III) |
| Version and Date: | 4.3 of 28.02.2013 |
| Sponsor: | Roche Pharma AG, Grenzach-Wyhlen |
| Objectives: | Indication: chronic hepatitis C  Medication: peg­interferon alfa-2a (PEGASYS), Ribavirin (COPEGUS)   - The main objective is to determine efficacy and safety of chronic hepatitis C treatment with peg­interferon alfa-2a (Pe­ga­sys®) – usually in combination with ribavirin (Cope­gus®) – in patients treated by gastroenterology specialists in real-life setting. A descriptive comparison of the col­lected data with existing safety data and data from the approval of peg­interferon alfa-2a (Pegasys®) alone or in combination with ribavirin (Copegus®), will further establish the feasibility of the products in day-to-day practice. - In addition, information on hepatitis C therapeutic habits and patient compliance will be gained. Quality of treatment will be assessed by comparing data from clinical studies with data from physicians’ practice. Furthermore, the quality of treatment in physicians’ practices and hospital outpatient departments will be contrasted. - Direct measures will be developed –in accordance with existing treatment recommendations (SPC, consensus recommendations) – to improve the quality of care for patients with hepatitis C. These measures are training courses, direct notifications on guideline-conform treatment during the documentation of patient data, periodic analyses of data to determine the current status, and, if great deviations from the recommended therapy are observed, by project participant’s supervision. - Cause analyses and analyses of errors made by participating physicians in practices or outpatient departments will be performed to optimize the overall support quality for the therapy of hepatitis C with peg­interferon alfa-2a (Pegasys®) and ribavirin (Copegus®).   Data will be systematically collected to further refine the safety profile of peg­interferon alfa-2a (Pegasys®) and ribavirin (Copegus®). |
| Selected patient population: | Therapy data from patients over 18 years of age, with a diagnosed chronic hepatitis C with detectable virus replication and no contraindication for the use of peg (40kDa)-interferon alfa-2a (Pegasys®) or combination therapy with ribavirin (Copegus®), can be documented (please see product information).  Women of childbearing potential should use adequate contraception. It is important that female patients of childbearing potential and their sexual partners use 2 contraceptive methods at the same time during treatment and 4 months after treatment discontinuation. During this time pregnancy tests have to be performed monthly.  Particular attention is also required and pregnancy should be avoided 7 months after treatment discontinuation in female sexual partners of male patients taking ribavirin (Copegus®). Both should use adequate contraception.  Ribavirin (Copegus®) must not be used in pregnant women.  Please see the current German product information. |
| Planned number of patients: | 9000 |
| Selected physician population: | Participation is open to all physicians familiar with interferon therapy in Germany. |
| Planned number of centers: | 600 |
| Selection criteria: | Selection will take place by a specialized field force. Criteria are experience of physicians in the treatment of chronic hepatitis C and a known excellent documentation quality in the past |
| Main parameters of interest: | Demographic data, anamnesis of hepatitis C, viral genotype and viral load, relevant laboratory parameters, histology (if available), concomitant diseases, data of treatment and dosage of Peginterferon alfa-2a and ribavirin and other concomitant medication, virological outcome: RVR, EVR, EOT, SVR, (severe) adverse events |
| Procedures: | Baseline, routine visits at weeks 2, 4, 8, 12, 24, 36, 48, EOT and EoFU, additional modules for HIV-coinfection, opioid substitution, cardiovascular risk, HCV after liver transplantation, and pharmacoeconomics |
| Statistical considerations: | It is planned to document at least 9000 cases in practices specialized in treatment of hepatitis and hospital outpatient departments, over a recruitment period of at least 4 years.  In Germany, 16,000 patients per year are treated with interferon therapy in practices specialized in hepatitis treatment, outpatient departments and hepatology units in hospitals. Thus, a sample size of approximately 12.5 % of all treated patients can be achieved. The sample is considered to be representative, taking into account the areal distribution of gastroenterologists in Germany. |
| Duration: | According to the product information, the duration of the combination therapy with ribavirin (Copegus®) for chronic hepatitis C depends on the virus genotype. Naive patients with virus genotype 1 and genotypes 2/3 should be treated for 48 weeks and 24 weeks, respectively. Patients with low viral load at baseline (< 800,000 IU/ml) and Rapid Viral Response (HCV-RNA undetectable after 4 weeks of treatment), can sufficiently be treated for 24 weeks only, in case of genotype 1, and for 16 weeks only, in case of genotypes 2/3.  This recommendation is identical for patients with genotype 4.  Duration of retreatment in patients who have failed prior therapy should be 48 weeks in general. In case of genotype 1 patients with prior non-response, retreatment duration of 72 weeks is recommended.  The recommended duration of Pegasys monotherapy is 48 weeks.  In clinical studies with peg­interferon alfa-2a (Pegasys®) alone or in combination with ribavirin (Copegus®) (see product information), a Rapid Viral Response was defined by a HCV RNA load below the limit of detection after 4 weeks of therapy whereas an early virological response was defined by a HCV RNA load below the limit of detection or by a reduction of the virus titre by 99 % of the initial value after 12 weeks of therapy.  Of patients with genotype 1 or 4 and no early virological response after 12 weeks therapy with peg­interferon alfa-2a (Pegasys®) alone or in combination with ribavirin (Copegus®), only a very small number (<2%) achieved sustained virological response.  In those patients, therapy should be discontinued, taking into account the low probability of a sustained virological response.  In patients with genotype 2 or 3, early virological response is known to be very high (97 %) and subsequently a high SVR rate is achieved after 24 weeks. Therefore, viral load determination after 12 weeks of treatment is not deemed to be necessary.  Planned FPI: 01/01/2008  Planned LPI: 31/12/2011  Planned LPO: 30/06/2013 |

# Objectives and rationale of the noninterventional study

## Background

### Introduction

The legislator rules that experience about the use of a medicinal product is to be systematically recorded and analyzed after the product has been approved (AMG Section 4 (23)). The resulting information about the occurrence of rare adverse drug reactions contribute greatly to drug safety. The data obtained can also provide valuable information about the actual, non-study-related use of the medication in the indication concerned as a form of quality assurance. The NIS data are therefore published in a recognized scientific journal after their scientific evaluation.

### Quality assurance in medicine

Quality assurance has at all times been a shared task of the medical community inherent in the practice of medicine. This was emphasized and reaffirmed at the 101^st^ German Medical Assembly in 1998 in relation to the principles of quality of assurance in the exercise of the medical profession already adopted in 1993.

The joint comments of the German Medical Association and the National Association of Statutory Health Insurance Physicians in response to the inquiry of the Conference of Ministers of Health of July 1998 also refer, among other things, to the following principles of quality assurance:

(Point 3) The sole purpose of quality assurance is to secure and improve patient
care and is therefore not an end in itself.
(Point 6) Quality assurance presupposes valid data and close cooperation between all concerned.
(Point 7) Quality assurance is not primarily research, but a target oriented, innovative ongoing and interdisciplinary process in all areas of medical care. It utilizes scientific methods to develop and evaluate suitable measures for use in hospitals and medical practices.
(Point 8) Quality assurance must not be confused with measures to improve economy in the health system, even though an improvement in cost effectiveness can be achieved with quality assurance methods.

Endeavours to improve quality management in the health service should therefore be directed primarily towards supporting the activities of the service providers. To systematically achieve a high quality of results, in view of the increasing abundance of information in medicine and medical care, the physician requires – in addition to his/her own observations – support in order to "do the right thing at the right time at the right place" when addressing a specific situation.

The main components of a quality management system of this type which aims to continuously improve the care process are described in the "Quality Management Guideline for Contractual Medical Care" of the JFC (Joint Federal Committee) which came into force on January 1, 2006. In Section 2 it provides, among other things – as the objective of an internal institutional quality management system – the objective demonstration and measurement of the results of care. In Section 4 relating to the instruments of an internal institutional QMS, reference is made, among other things, specifically to the documentation of courses of treatment and advisory activities.

It is therefore logical that partners in the health service – physicians on the one side, medicinal product manufacturers on the other side – should jointly develop a documentation system in the form of an online noninterventional study with the aim of realistically representing the care of chronically ill patients with simultaneous controlling of technical drug safety aspects of the medications used.

### Hepatitis C

Chronic virus hepatitis C is a disease which often takes a clinically insidious course and threatens the affected patients mainly because of its late complications (liver cirrhosis, hepatocellular cancer). In June 2002, peg­interferon alfa-2a (Pegasys®) was approved for the treatment of chronic virus hepatitis C by the European Agency for the Evaluation of Medicinal Products (EMEA) on the basis of two international marketing authorization studies (Zeuzem et al. 2000, Heathcote et al. 2000).

In patients with chronic hepatitis C, Pegasys® is best used in combination with ribavirin.

Since January 2003, ribavirin Copegus® has been approved by the Federal Institute for Drugs and Medical Devices (BfArM) in Germany for combination with peg­interferon alfa-2a (Pegasys®) in the treatment of chronic hepatitis C.

The combination of Pegasys® and ribavirin is indicated in non-pretreated patients and in patients who have failed to respond to previous treatment with interferon alfa (pegylated or non-pegylated) alone or in combination therapy with ribavirin.

Monotherapy with peg­interferon alfa-2a is indicated mainly when there is intolerance or contraindications to ribavirin.

Pegylated interferon alfa-2a (Pegasys®) is administered to patients with genotype 1 and the more rarely occurring genotypes 4, 5 and 6 in the combination with ribavirin initially for 12 weeks. The decision whether to prolong the therapy by 36 weeks to altogether 48 weeks or to discontinue the treatment is based on an HCV-RNA assay: either the virus assay in week 12 is negative (equivalent to a reduction in the viral load below the limit of detection) or a reduction was achieved in the original serum HCV-RNA by 2 log steps (equivalent to 99 % of the baseline value before start of treatment).

Patients who did not show this defined "early virological response" (EVR) after 12 weeks will with very high probability (97 %) also not have any lasting therapeutic success after further treatment (Ferenci P et al. 2001, Fried MW et al. 2001). This procedure saves the patient unnecessary stress from treatment side effects and saves costs.

In patients with genotypes 2 or 3, on the other hand, such a high EVR of 97 % is achieved that no monitoring of the viral load after 12 weeks is recommended.

As regards the duration of treatment, a further multicentre international study for patients with genotypes 1 and 4 also confirmed a treatment duration of 48 weeks (Hadziyannis SJ et al. 2004).

Treatment over 24 weeks may be considered for patients showing an infection with genotype 1 and a low initial viral load (LVL) (≤ 800,000 IU/ml) or with genotype 4 or who become HCV-RNA negative (≤ 50 IU/ml) by week 4 and remain HCV-RNA negative up to week 24 (Jensen D et al. 2006, Ferenci et al. 2006).

A total treatment period of 24 weeks, however, may be associated with a higher risk of relapse than treatment over 48 weeks. In these patients, the tolerability of the combination therapy as well as prognostic factors such as the degree of fibrosis should be taken into account when deciding the duration of treatment. In patients with a genotype 1 infection and a high initial viral load (HVL) (>800,000 IU/ml), and who become HCV-RNA negative by week 4 and remain HCV-RNA negative up to week 24, a shortening of the treatment duration should nevertheless be considered with caution because the limited data available suggest that shortening the treatment in this patient population may have a significantly negative impact on the persistent virological response.

Patients with genotypes 2 and 3 are normally treated with a duration of 24 weeks. No difference in efficacy was observed compared to a 48-week treatment.

A shortened treatment for 16 weeks may be considered for such genotype 2/3 patients who show a low initial viral load (LVL) (≤ 800,000 IU/ml) and who are already HCV-RNA negative in week 4 (≤ 50IU/ml) and remain HCV-RNA negative up to week 16.

As with the possibility of shortening the treatment period for genotype 1, the tolerability and prognostic factors should also be taken into account with genotype 2/3 when deciding on a reduction of the treatment period.

For patients with genotypes 5 and 6 less well substantiated by studies, the same recommendations are given as for the genotype 1 with HVL.

Patients with HIV-HCV coinfection should be treated over 48 weeks regardless of the genotype. A treatment period of less than 48 weeks has not been adequately researched.

The recommended total duration for retreatment of previously treated patients is 48 weeks. Patients with a detectable viral load in week 12 of the retreatment should discontinue this retreatment.

If retreatment is considered for patients who are infected with virus genotype 1 and who fail to respond to previous treatment with pegylated interferon plus ribavirin, the recommended total duration of treatment is 72 weeks.

The following information on the dosage of ribavirin (Copegus®) in combination with peg­interferon alfa-2a (Pegasys®) can also be derived from the study of Hadziyannis et al.:

Patients with genotype 1 with a body weight of less than 75 kg should receive 1000 mg ribavirin (Copegus®) orally per day, and with a body weight above 75 kg should receive 1200 mg. This recommendation has so far also applied to the interferon-ribavirin combination treatment of patients with genotype 2 or 3. The current data now show that patients with genotype 2 or 3 require only 800 mg ribavirin (Copegus®) orally per day regardless of body weight if administered in combination with peg­interferon alfa-2a (Pegasys®). This change in the recommendation for the ribavirin dosage on combination with peg­interferon alfa-2a (Pegasys®) can reduce unnecessary stress from treatment side effects and can also lower costs.

Further international studies have demonstrated the high efficacy and good safety profile of peg­interferon alfa-2a (Pegasys®) in combination with ribavirin (Copegus®) in patients with normal GPT (Zeuzem et al. 2004) and in HIV/HCV coinfected patients (Torriani et al. 2004).

These findings have meanwhile been incorporated in numerous national guidelines, including the German guidelines, as well as recommendations of international associations for liver research (EASL, AASLD).

## Formulation of the research question

In part I and II of the observational study (ML17071 and ML19464) the actual condition of patient care was documented and evaluated. The large number of documented patients also made it possible to describe and analyze patient populations which previously had been represented only inadequately in studies and for whom only data from very small cohorts are available. (Hüppe et al. 2004, Hüppe et al. 2005, Hüppe et al. 2006 Hüppe et al. 2007, Meyer et al. 2007, Zehnter et al. 2004, Zehnter et al. 2005, Zehnter et al. 2006, Zehnter et al. 2007). However, it is found to be more favourable to modify the recording of certain data in such a way that statements of future analyses can provide more precise presentations than today.

The basic objectives of this noninterventional study have not changed in relation to ML17071 and ML19464.

Specifically, these are:

- As the main objective, the efficacy and tolerability of the treatment of chronic hepatitis C with peg­interferon alfa-2a (Pegasys®), usually in the combination with ribavirin (Copegus®), are studied in patients being treated by gastroenterologists and other physicians familiar with interferon therapy in specialized practices and hospital outpatient departments in the real-life setting.
  The everyday applicability of the study results can be verified by a descriptive comparison of the recorded data with the efficacy and tolerability data from clinical studies on peg­interferon alfa-2a (Pegasys®) alone or in combination with ribavirin (Copegus®).
- Knowledge is to be generated about the therapeutic habits in hepatitis C and about patient compliance. This is to allow a statement to be made regarding the quality of treatment of patients with hepatitis C in Germany. Conclusions are also to be reached regarding the quality of treatment in residential practice, expressed in relation to the data from hospital outpatient departments.
- Based on the treatment quality data, measures which lead to an improvement in the quality of care of patients with hepatitis C are to be developed in accordance with the existing therapy recommendations (Summary of Product Characteristics, consensus recommendations of the medical societies, recommendations based on recent study findings). These measures consist of further training events, provision of direct information about guideline compliant treatment of the patient during the internet assisted data documentation, regular analyses of the data to determine the current status and, if there are considerable deviations from the recommendations, treatment by supervising the project participants.
- In addition, root cause and error analyses are to be performed for individual participating medical practices or hospital outpatient departments to selectively optimize the quality of care in the treatment of hepatitis C with peg­interferon alfa-2a (Pegasys®) and ribavirin (Copegus®).
- Safety data on peg­interferon alfa-2a (Pegasys®) and ribavirin (Copegus®) are recorded systematically and matched against the known safety profile.

Medicinal product used in standard treatment of hepatitis C:

Pegylated (40 kDa) Interferon alfa-2a (Pegasys®)

Marketing Authorization Holder: Roche Pharma AG, Grenzach-Wyhlen (Germany)

Marketing authorization number:

EU/1/02/221/005 PEGASYS 135 microgram injection solution in a prefilled syringe in 0.5 ml solution – 1 prefilled syringe + 1 injection needle

EU/1/02/221/006 PEGASYS 135 microgram injection solution in a prefilled syringe in 0.5 ml solution – 4 prefilled syringes + 4 injection needles

EU/1/02/221/009 PEGASYS 135 microgram injection solution in a prefilled syringe in 0.5 ml solution – 12 prefilled syringes + 12 injection needles

EU/1/02/221/007 PEGASYS 180 microgram injection solution in a prefilled syringe in 0.5 ml solution – 1 prefilled syringe + 1 injection needle

EU/1/02/221/008 PEGASYS 180 microgram injection solution in a prefilled syringe in 0.5 ml solution – 4 prefilled syringes + 4 injection needles

EU/1/02/221/010 PEGASYS 180 microgram injection solution in a prefilled syringe in 0.5 ml solution – 12 prefilled syringes + 12 injection needles

54828.00.00 Copegus 200 mg film-coated tablets, HDPE bottles with 28, 42, 112 or 168 tablets

66105.00.00 Copegus 400 mg film-coated tablets, HDPE bottles with 14 or 56 tablets

The current Summary of Product Characteristics is enclosed with the documentation.

## Rationale for choice of method

This trial is therapeutically and diagnostically noninterventional. The procedure followed by the involved physicians is not influenced by this trial. The physician is completely free in his/her decision regarding which patients he/she treats with the medication observed in this trial, which doses he/she chooses, which diagnostic measures he/she takes, how he/she monitors the course of treatment or which concomitant or additional medication he/she prescribes. The appointments for the physician-patient contacts are determined individually. The documentation time points are scheduled.

The choice of this methodological approach in the noninterventional study necessarily follows from the subject of investigation, which is to collect scientific data reflecting the clinical routine as far as possible.

Recording of data on the course and outcome of treatment in daily medical practice can only reflect reality in a noninterventional study. The decisive criterion for this is the inclusion of a large number of gastroenterology or other specialized practices and hospital outpatient departments experienced with interferon treatment, which are deliberately not chosen to comply with the strict requirements of a clinical study and which routinely treat hepatitis C patients. Moreover, access to patients is on an open basis; data are recorded for patient populations that reflect a majority of the hepatitis C patients in Germany, but who are normally not investigated in clinical studies because of strict inclusion and exclusion criteria (e.g. addiction patients or HIV-HCV coinfected patients).

# Selection criteria

## Procedure for selection of participating physicians

Any physician providing routine treatment with peg­interferons to HCV infected patients can participate in the noninterventional study. In Germany, HCV treatment is delivered mainly in specialized practices and hospital outpatient departments. These sites are specifically chosen for the noninterventional study by the field force of Roche Pharma AG, Grenzach-Wyhlen.

It is planned to included around 600 centres in the prospective noninterventional study.

## Description of patient access

Reflecting the research subject of this noninterventional study, the members of the patient population generally suffering from chronic hepatitis C are included in this study who require medicinal treatment during therapy and for whom the treating physician takes the individual decision to provide treatment with peg­interferon alfa-2a (Pegasys®).

The treating physician is requested to document the treatment data of all patients for whom he/she takes the individual decision to perform the medicinal therapy with peg­interferon alfa-2a (Pegasys®) based on therapeutic necessity in the period from 1 January 2008 to 31 December 2011 after receiving the study documentation.

# Conduct of the noninterventional study

## Duration and premature termination of the noninterventional study

The noninterventional study will start on 1-Jan-2008 and is scheduled to end on 30-Jun-2014. The individual duration of the documentation of a patient is about 72 weeks.

The noninterventional study is intended to cover the entire treatment period (depending on the physician's decision and the main baseline factors such as genotype and viral load, or up to the end of treatment with peg­interferon alfa-2a (Pegasys®). Independently of the treatment decision taken, concluding documentation is to be generated for every patient even after discontinuation of treatment with peg­interferon alfa-2a (Pegasys®).

In the event of premature termination of the overall prospective noninterventional study, for example for regulatory reasons, the available data will be analyzed completed and a final report of the prospective noninterventional study will be compiled, if appropriate in a reduced form.

## Procedure

This trial does not influence the individual course of treatment. The noninterventional study has no influence on the medical decisions and procedures. The participating physician is requested, however, to document the decisions and measures taken in the case record form of this noninterventional study.

The documents attempt to reflect the usual course of treatment as closely as possible and thereby facilitate documentation. Under no circumstances are they to be misunderstood as instructions for the management of treatment.

The documentation procedure per patient is as follows:

1. the diagnosis is made and the treatment decision is taken outside the study
2. patient informed consent
3. patient status and statement regarding start of study
4. any interim findings
5. patient status and information on treatment at end of study. The final documentation should generally be performed 48 – 72 weeks after the start of the study or at the end of the treatment, if this is sooner.

# Description of nature and scope of documentation

## General structure of the case record form

Before inclusion in the noninterventional study, the patient and the participating physician sign an informed consent form (see Annex 14.1). This remains with the treating physician and is not part of the case record form.

An electronic case record form must be completed for every patient participating in the noninterventional study and must be available with an electronic signature of the physician or a person authorized by him/her. This also applies for patients in whom an initial treatment was commenced and who may have been switched to a different treatment.

Since the documentation in a noninterventional study should be prospective, the participating physician is requested to ensure that data documentation is generated as soon as possible. The software for data recording in the noninterventional study is therefore programmed so that dates older than three months are not accepted.

Patient names must not be shown on the case record form or in other documents forwarded to the sponsor.

The physician or a person authorized by him/her receives a user name and a password for access to enter data into online electronic case record forms. Entry is completed and confirmed by the corresponding confirmation at the end of each case record form.

All information recorded in the noninterventional study is confidential. If the participating physician makes the records of the noninterventional study accessible to a third party or transfers them to a different location, Roche Pharma AG, Grenzach-Wyhlen is to be informed in writing in advance.

## Survey variables

The case record forms allow the recording of laboratory values and clinical findings usually recorded during the diagnostic procedures for virus hepatitis.

The headings provided for this purpose do not mean that all parameters need necessarily be investigated.

If the data are recorded, however, they should be documented completely in order to increase the evidential value of this noninterventional study.

The following parameters can be documented in this observational study:

### Practice characteristics (multiple responses possible)

– These data are updated every six months:

- Member of the Association of German Resident Gastroenterologists (BNG) yes/no
- Type of facility
- single, group practice, specialized practice, hospital outpatient department, university hospital outpatient department
- Physicians in the participating practice/outpatient department:
- general practitioner, internal specialist, gastroenterologist, hematologist / oncologist, infectious diseases specialist, addiction medical specialist, psychiatrist, other.
- Number of patients cared for last year with:
- HCV, HBV or HIV infection, HIV/HCV and HIV/HBV coinfection, opioid substitution, post liver transplantation
- Regular cooperation with:
- psychiatrists / psychologists / ext. DCC
- internal specialists, surgeons, institutes/outpatient departments, detoxification

Physicians performing substitution:

- Regular monitoring of concomitant use:
- alcohol monitoring / quarterly, visual urine testing / quarterly
- Patient substitution status (number):
- methadone, buprenorphine, polamidone, other
- Maintenance, attitude towards cessation, detoxification (absolute or in %)

### Main module for recording treatment of hepatitis C

Important: parameters of particular importance in various modules are also presented there. To prevent documentation errors, such values are presented in all modules independently of the module in which they are entered.

#### Baseline examination

- Written patient informed consent (date)
- Patient data for age, gender, height, weight (BMI is calculated automatically from the latter two parameters), ethnicity, speech communication and first language learned (German, Turkish, Russian, Polish, Italian, other)
- Patient's insurance status: statutory health insurance, private health insurance, self-pay patient
- First three characters of the postal code of the patient's residence
- Concomitant diseases

| Psychiatric illness e.g. depression, psychosis, status post attempted suicide, currently with/without treatment | Inflammatory bowel disease |
| --- | --- |
| Cardiovascular: arterial hypertension, CHD/angina pectoris, myocardial infarction, PAOD | Hepatitis A |
| Malignant diseases: Hodgkin, non-Hodgkin (incl. lymphatic leukemia), skin tumours | Hepatitis B |
| Diabetes mellitus | Acquired immune weakness (AIDS/HIV)*: anti-HIV-IgM |
| Chronic respiratory tract diseases: asthma, COPD | Thyroid dysfunction: hypofunction hyperfunction |
| Inflammatory joint disease (including rheumatism) | Arterial vascular disease |
| Lipid metabolism disorders | Skin disease psoriasis  neurodermatitis |
| Alcohol abuse / dependence | Syphilis |
| Drug abuse: active drug misuse (intravenous / other) drug substitution therapy (methadone, polamidone, buprenorphine, heroin, other) status post drug misuse | TBC |
| Liver function disorder | Herpes zoster |
| Renal insufficiency | Dental and peridental diseases, e.g. caries |
| Lipid distribution disorder | Allergies |
| Ophthalmological diseases e.g. retinopathies, retinal vascular occlusions | Status post organ transplantation: liver, kidney lung, heart |
| Neurological diseases e.g. convulsions | Other |
| Polyneuropathy |  |

- Route of transmission of hepatitis (mode of infection): blood products, illicit drugs (intravenous / nasal), sexual transmission, tattooing / piercing, needle stick injury, surgical / medical intervention, medical / dental staff, dialysis, unknown, other

Medical history: naive;
 Relapser/nonresponder to previous treatment with: IFN monotherapy
 IFN-RBV combination therapy

PEG monotherapy
 PEG-RBV combination therapy
 Reason for relapse / nonresponse: virological nonresponse
 inadequate previous treatment
 intolerability

- Estimated duration of infection:
- Clinical symptoms / laboratory values at first diagnosis that led to the diagnosis "chronic hepatitis C": none, fatigue, fever, muscular stiffness, skin changes, hair loss, joint pain, muscle pain, abdominal pain, nausea, reflux, weight loss, headache, insomnia, irritability, restlessness, depression, depressive mood, psychosis, impaired concentration, anorexia, jaundice, pruritus, elevated GPT, craving / withdrawal-like symptoms, other:
- Laboratory parameters
  GPT (=ALT), GOT (= AST), gamma-GT
  Other laboratory parameters:
  Hemoglobin, RBC, platelets, WBC, neutrophils (%), TSH, Quick's value, AP, bilirubin, creatinine, serum ferritin, serum iron, transferrin, alpha fetoprotein, albumin, blood glucose (fasting), HbA1c, triglycerides, cholesterol (total), HDL, LDL, uric acid, CRP quantitative.
- Hepatitis C laboratory diagnostic tests:
  HCV-PCR (qualitative/quantitative)
  Anti-HCV
- HCV genotype
- Hepatitis B laboratory diagnostic tests: HBsAg (+/-), anti-HBc (+/-), if +, then anti-HBc-IgM; if HBsAg+, then HBeAg +/-; HBV-DNA quant./qual., HBV genotype, anti-HBs (+/-); anti-HDV (+/-), if +, then HDV-RNA (+/-, qual. or quant.)
- Autoantibodies: ANA, LKM, SMA, AMA, SLA, Trak, MAK, rheumatoid factors, other
- Vaccination status/vaccination protection against hepatitis A and B: yes/no
- Clinical examination:
  – ultrasound: normal findings, splenomegaly, picture of chronic liver disease, fatty liver, sonographic signs of liver cirrhosis, result unknown since performed by physician providing previous care
  – histology: date performed; stage of fibrosis and degree of inflammatory activity after selection of score
  – fibroscan (kPa)
- Clinical findings:
  Normal findings, spider naevi, cirrhosis (Child-Pugh classification), hepatic encephalopathy, esophageal varices, ascites
- Alcohol consumption no/yes, if yes, then inquiry ≤ / > 40 g/day and drinks/day in the last 30 days
- Assessment of the patient's quality of life impairment by hepatitis C: none, slight, moderate, severe
- Questions on repeated documentation in this or a previous online observational study in the BNG
- Start of treatment
- Initial dose of peg­interferon alfa-2a (Pegasys®): 180 µg, other dosage
- Treatment with ribavirin, e.g. Copegus®: 800, 1000, 1200 mg/day, other dose, dose schedule
- Concomitant therapy
- Submission of the SF-36 and sociodemography record form before therapy (questionnaire to be completed by the patient)
- Pregnancy test
- Contraception

#### Examination after 2 weeks

- Laboratory parameters
  GPT (=ALT), GOT (= AST), GGT
  Other laboratory parameters:
  hemoglobin, platelets, WBC, neutrophils (%), bilirubin
- Adverse events (including suspected cases) with substance-specific causality survey
- Date and dosage information for peg­interferon alfa-2a (Pegasys®): 180 µg and ribavirin, e.g. Copegus® (including dose changes and rationale for change, dose schedule)
- Concomitant medication
- New comorbidities
- Clinical symptoms: none, fatigue, fever, muscular stiffness, skin changes, hair loss, joint pain, muscle pain, abdominal pain, nausea, reflux, weight loss, headache, insomnia, irritability, restlessness, depression, depressive mood, psychosis, impaired concentration, anorexia, jaundice, pruritus, elevated GPT, craving / withdrawal-like symptoms, other;
  causal relationship with Pegasys and/or ribavirin
- Treatment-related sick leave (duration) since the last examination
- Pregnancy test

#### Examinations during treatment in week 4, 8, 12, 24, 36 and 48

- *Only in week 12, 24, 36 and 48:* body weight
- Laboratory parameters
  GPT (=ALT), GOT (= AST), GGT,
  Other laboratory parameters:
  hemoglobin, platelets, WBC, neutrophils (%), bilirubin, creatinine
  *Only in week 12, 24, 36 and 48*: TSH, blood glucose (fasting), HbA1c *Only in week 24:* ANA, LKM, SMA, AMA, SLA, Trak, MAK, rheumatoid factors
- Hepatitis laboratory diagnostic tests
  HCV-PCR (quantitative/qualitative), *in week 4 and 12 recommended*
  HCV-PCR (qualitative), *only in week 24* for GT-2/3, o*nly in week 48 for GT-1/4 recommended*
  (HCV-PCR (quantitative/qualitative) can be documented in week 4, 8, 12, 24 and 48)
- Adverse events (including suspected cases) with substance-specific causality survey
- Date and dosage information for peg­interferon alfa-2a (Pegasys®): 180 µg and ribavirin, e.g. Copegus®: (including dose changes and rationale for the change, dose schedule)
- Concomitant medication
- New comorbidities
- Clinical symptoms: none, fatigue, fever, muscular stiffness, skin changes, hair loss, joint pain, muscle pain, abdominal pain, nausea, reflux, weight loss, headache, insomnia, irritability, restlessness, depression, depressive mood, psychosis, impaired concentration, anorexia, jaundice, pruritus, elevated GPT, craving / withdrawal-like symptoms, other;
  causal relationship with Pegasys and/or ribavirin
- Treatment-related sick leave (duration) since the last examination
- (Monthly) pregnancy test
- *Only in week 12, 24, 36 and 48:*
  Clinical assessment of the tolerability of Pegasys® and the combination with ribavirin (Copegus®): very good, good, moderate, poor,
- *Only in week 12, 24, 36 and 48:*
  Compliance (assessed by the physician):
   very good: all medications taken
   good: max. 10% not taken
   satisfactory: max. 20% not taken
   poor: > 20% not taken
- *Only in week 12, 24, 36 and 48:*
  Quality of life assessment compared to before treatment: symptom-free; improved, but not symptom-free; unchanged
- *Only in week 12, 24, 36 and 48:*
  Alcohol consumption no/yes, if yes, inquiry ≤ / > 40 g/d and drinks/d in the last 30 days
- *Only in week 12:* *Submission of the SF-36 and sociodemography record form (questionnaire to be completed by the patient)*

#### At the end of treatment / documentation (end of therapy):

- Therapeutic consequence: reason for end / discontinuation of therapy
- Reasons for therapy discontinuation: lacking virological response, lacking tolerability, lacking compliance, unplanned event (e.g. imprisonment, detoxification), patient's wish, patient no longer attended, other reason
- Concomitant medication
- New comorbidities
- Body weight
- Hepatitis C laboratory diagnostic tests
  HCV-PCR (quantitative/qualitative), *only recommended in week 12*
  HCV-PCR (qualitative), *only recommended in week 24 for GT-2/3, only in week 48 for GT-1/4*
  (HCV-PCR (quantitative/qualitative) can be documented in week 4, 8, 12, 24 and 48)
- Hepatitis B laboratory diagnostic tests (only if chronic hepatitis B was documented at the baseline examination): HBsAg (+/-), HBeAg +/-; HBV-DNA quant./qual., anti-HBs (+/-); anti-HDV (+/-), HDV-RNA (+/-, qual. or quant.)
- Autoantibodies: ANA, LKM, SMA, AMA, SLA, Trak, MAK, rheumatoid factors, other
- Laboratory parameters
  GPT (=ALT), GOT (= AST), GGT,
  Other laboratory parameters:
  hemoglobin, platelets, WBC, neutrophils (%), bilirubin, creatinine, TSH, blood glucose (fasting), HbA1c, triglycerides, cholesterol, HDL, LDL, serum ferritin
- Clinical symptoms: none, fatigue, fever, muscular stiffness, skin changes, hair loss, joint pain, muscle pain, abdominal pain, nausea, reflux, weight loss, headache, insomnia, irritability, restlessness, depression, depressive mood, psychosis, impaired concentration, anorexia, jaundice, pruritus, elevated GPT, craving / withdrawal-like symptoms, other;
  causal relationship with Pegasys and/or ribavirin
- Treatment-related sick leave (duration) since the last examination
- (Monthly) pregnancy test
- Adverse events (including suspected cases) with substance-specific causality survey
- Clinical assessment of the tolerability of Pegasys® and the combination with ribavirin (Copegus®): very good, good, moderate, poor
- Quality of life assessment compared to before treatment: symptom-free; improved, but not symptom-free; unchanged
- Alcohol consumption no/yes, if yes, inquiry ≤/ > 40 g/d and drinks/d in the last 30 days
- Compliance (assessed by the physician):
   very good: all medications taken
   good: max. 10% not taken
   satisfactory: max. 20% not taken
   poor: > 20% not taken
- Quality of life assessment compared to before treatment: symptom-free; improved, but not symptom-free; unchanged
- Submission of the SF-36 and sociodemography record form (questionnaire to be completed by the patient)

#### To week 24 of follow-up

- Body weight
- Laboratory parameters
  GPT (=ALT), GOT (= AST), GGT,
  Other laboratory parameters:
  hemoglobin, platelets, WBC, neutrophils (%), bilirubin, creatinine, TSH, blood glucose (fasting), HbA1c, triglycerides, cholesterol, HDL, LDL, serum ferritin ANA, LKM, SMA, AMA, SLA, Trak, MAK, TSH, rheumatoid factors
- Hepatitis C laboratory diagnostic tests
  HCV-PCR (qualitative/quantitative) *only qualitative recommended*
- Hepatitis B laboratory diagnostic tests (only if chronic hepatitis B was documented at the baseline examination): HBsAg (+/-), HBeAG +/-; HBV-DNA quant./qual., anti-HBs (+/-); anti-HDV (+/-), HDV-RNA (+/-, qual. or quant.)
- Adverse events (including suspected cases) with substance-specific causality survey
- Concomitant medication
- Clinical symptoms: none, fatigue, fever, muscular stiffness, skin changes, hair loss, joint pain, muscle pain, abdominal pain, nausea, reflux, weight loss, headache, insomnia, irritability, restlessness, depression, depressive mood, psychosis, impaired concentration, anorexia, jaundice, pruritus, elevated GPT, craving / withdrawal-like symptoms, other treatment-related sick leave (duration) since the last examination
- Pregnancy
- Quality of life assessment compared to before treatment: symptom-free; improved, but not symptom-free; unchanged
- Alcohol consumption no/yes, if yes, inquiry ≤/ > 40 g/d and drinks/d in the last 30 days
- Submission of the SF-36 and sociodemography record form (questionnaire to be completed by the patient)

### Additional module for recording treatment of hepatitis C in opioid substituted patients

#### Baseline examination

- Addiction history (acc. to EuropASI)
- Substitution therapy: methadone, L-polamidone, buprenorphine, buprenorphine/naloxone combination, heroin, other, in each case stating the dose/day;
  on-site in the practice, in pharmacy: daily, how often per week / take-home, how often per week
- Urinalysis: no/yes, if yes then: -/+: cocaine, benzodiazepines, barbiturates, amphetamines, cannabis, methadone, L-polamidone, buprenorphine, heroin, EDDP, creatinine, if positive, then inquire about measured value
- Laboratory parameters:
  MCV, CRP quant., TPHA (syphilis), Mendel-Mantoux test (tuberculosis)
- Psychosocial care ( most probable):
- 1h/day, 1-2h/week, 1-2h/month, feedback 1x/quarter
- done by: substituting physician him/herself, qualified social education worker, psychologist, other specialist
- on-site in the practice, externally in DCC

#### Examination after 2 weeks

- Urinalysis: no/yes, if yes then: -/+: cocaine, benzodiazepines, barbiturates, amphetamines, cannabis, methadone, L-polamidone, buprenorphine, heroin, EDDP, creatinine, if positive, then inquire about measured value
- Substitution therapy: methadone, L-polamidone, buprenorphine, buprenorphine/naloxone combination, heroin, other, in each case stating the dose/day;
  on-site in the practice, in pharmacy: daily, how often per week / take-home, how often per week

#### Examinations during the treatment at week 4, 8, 12, 24, 36 48, at end of hepatitis C therapy/documentation

- Urinalysis: no/yes, if yes then: -/+: cocaine, benzodiazepines, barbiturates, amphetamines, cannabis, methadone, L-polamidone, buprenorphine, heroin, EDDP, creatinine, if positive, then inquire about measured value
- Substitution therapy: methadone, L-polamidone, buprenorphine, buprenorphine/naloxone combination, heroin, other, in each case stating the dose/day;
  on-site in the practice, in pharmacy: daily, how often per week / take-home, how often per week
- ***Only in week 24, 48 and at the end of hepatitis C therapy/documentation:***Substance use (acc. to EuropaASI, restricted to the consumption behaviour in the last 30 days)
- ***Only in week 24 and 48:*** Psychosocial care (most probable):
- 1h/day, 1-2h/week, 1-2h/month, feedback 1x/quarter
- done by: substituting physician him/herself, qualified social education worker, psychologist, other specialist
- on-site in the practice, externally in DCC

#### At week 24 of follow-up

- Urinalysis: no/yes, if yes then: -/+: cocaine, benzodiazepines, barbiturates, amphetamines, cannabis, methadone, L-polamidone, buprenorphine, heroin, EDDP, creatinine, if positive, then inquire about measured value
- Substance use (acc. to EuropaASI, restricted to the consumption behaviour in the last 30 days)
- Substitution therapy: methadone, L-polamidone, buprenorphine, buprenorphine/naloxone combination, heroin, other, in each case stating the dose/day;
  on-site in the practice, in pharmacy: daily, how often per week / take-home, how often per week
  "Patient no longer receives substitution therapy (e.g. because patient is "clean")"
- Psychosocial care ( most probable):
- 1h/day, 1-2h/week, 1-2h/month, feedback 1x/quarter
- done by: substituting physician him/herself, qualified social education worker, psychologist, other specialist
- on-site in the practice, externally in DCC

### Additional module for recording therapy of hepatitis C in HIV coinfected patients

#### Baseline examination

At the start of treatment with peg­interferon alfa-2a (Pegasys®) (baseline), the following parameters were recorded for patients with detected HIV infection:

- First HIV diagnosis (year), if known
- Route of transmission of HIV (mode of infection): illicit drugs (intravenous), MSM, heterosexual, unknown, other.
- Stage of disease (CDC)
- Previous diseases: AIDS-defining events
- Antiretroviral pretreatment: ART-naive / previously treated (*excluding current ART*)

If previously treated: start of first ART (antiretroviral treatment)

- - The patient was previously treated with [number of] ART regimens.
  - The patient was already treated with the following ART classes.

NRTI

NNRTI

PI

FI

Integrase inhibitor

CCR5 antagonist

- Resistance situation (FASTA file)
- Clinical laboratory tests:
  HIV-RNA, CD4 / CD8 cell count (absolute and %, including following visits), LDL, HDL, VLDL (fasting)
- Antiretroviral treatment including treatment pauses and reasons for start, change or discontinuation
- Patient compliance with antiretroviral medication
- Tolerability of antiretroviral medication (signature tox.)
- Concomitant medication: lipid reducers, antimycotics, tuberculostatics, antdiarrheals, antacids

#### Examinations during treatment in week 12, 24 and 48, at the end of hepatitis C treatment/documentation and in week 24 of follow-up

- Antiretroviral treatment including treatment pauses and reasons for start, change or discontinuation
- Clinical laboratory tests:
  HIV-RNA, CD4 / CD8 cell count, LDL, HDL, VLDL (fasting)
- Resistance situation, if appropriate (FASTA file)
- Patient compliance with antiretroviral medication
- Tolerability of antiretroviral medication (signature tox.)
- Concomitant medication: lipid reducers, antimycotis, tuberculostatics, antdiarrheals, antacids

### Additional module to record treatment of hepatitis C in patients with cardiovascular risk

#### Baseline examination

- Metabolic syndrome

**Definition according to WHO (1999):**

*Glucose intolerance, impaired glucose tolerance (IGT) or diabetes mellitus and/or insulin resistance together with two or more of the other criteria listed below:*

Criteria:

- Impaired glucose regulation or diabetes
- Insulin resistance (highest quartiles of the HOMA-IR index)
- Dyslipidemia:

Triglycerides: ≥ 150 mg/dl (1.7 mmol/l) and/or

HDL cholesterol: ♂:< 35 mg/dl (0.9 mmol/l), ♀: < 39 mg/dl (1.0 mmol/l)

- Hypertension: ≥ 140/90 mm Hg and/or corresponding treatment
- Central obesity: waist-to-hip ratio: ♂: > 0.9, ♀: > 0.85 and/or BMI ≥ 30 kg/m^2^

Microalbuminuria: ≥ 20 µg/min or ≥ 30 mg/g creatinine (albumin-to-creatinine ratio)

or

**Definition acc. to NCEP_III_ATP (2001) synonym ATP III:**

Criteria:

- Hypertriglyceridemia: ≥ 150 mg/dl (1.7 mmol/l)
- Lowered HDL cholesterol: ♂:< 40 mg/dl, ♀: < 50 mg/dl
- Hypertension: ≥ 130/85 mm Hg and/or corresponding treatment
- Central obesity: BMI > 30 kg/m^2^ or waist: ♂: > 102 cm, ♀: >88 cm

Elevated fasting plasma glucose: ≥ 110 mg/dl (6.1 mmol/l)

or

**Definition acc. to IDF (2005):**

*Central obesity defined as: waist: ♂: > 94 cm (European), ♀: >80 cm (European) + 2 of the following criteria*

*Central obesity defined as: waist: ♂: > 90 cm (Asian), ♀: >80 cm (Asian) + 2 of the following criteria*

*Central obesity defined as: waist: ♂: > 85 cm (Japanese), ♀: >90 cm (Japanese) + 2 of the following criteria*

*For South and Central Americans the values for Asians are used.*

*For Africans, Mediterranean inhabitants and inhabitants of the Middle East the values for Europeans are used.*

Criteria:

- Hypertriglyceridemia: ≥ 150 mg/dl (1.7 mmol/l) or corresponding treatment
- Lowered HDL cholesterol: ♂:< 40 mg/dl (0.9 mmol/l), ♀: < 50 mg/dl (1.1 mmol/l) or corresponding treatment
- Hypertension: ≥ 130/85 mm Hg or corresponding treatment
- Elevated fasting plasma glucose: ≥ 100 mg/dl (5.6 mmol/l) or type 2 diabetes
- Diabetes mellitus type
- Overweight
- Blood pressure parameters
- Hip and waist circumference (calculation of waist-to-hip ratio)
- Smoker:
  1. currently smoking: patients who have previously smoked ≥ 100 cigarettes and are currently smoking.
  2. former smokers: patients who have previously smoked ≥ 100 cigarettes and are currently not smoking.
  Nonsmokers:
  patients who have previously smoked < 100 cigarettes.
- Laboratory parameters:
  Na, K, Ca, insulin (fasting)
  (Calculation of the HOMA-IR index:
  [fasting insulin (in mIU/l) x fasting plasma glucose (in mmol/l)/ 22.5]
  or
  [fasting insulin (in mIU/l) x fasting plasma glucose (in mg/dl)/ 405]),
  (repeat presentation of HbA_1c_, HDL, LDL, triglycerides, cholesterol (total), glucose (fasting), uric acid, BMI)
- Special concomitant medication

Diabetes: insulins and insulin analogues, oral antidiabetics (e.g. alpha-glucosidase inhibitors, biguanide derivatives, sulfonylurea derivatives, "glinides", "glitazones")

Hypertension: antihypertensives (e.g. ACE inhibitors, angiotensin II antagonists, centrally acting antihypertensives, beta blockers, calcium channel blockers, diuretics, combination products)

Dyslipidemia: lipid reducers (e.g. HMG-CoA reductase inhibitors, fibrates, nicotinic acid and derivatives,

Obesity: e.g. lipase inhibitors, appetite suppressants

- Familial history: diseases such as hypertension, myocardial infarction, stroke

#### Examinations during treatment in week 24 and 48, at the end of hepatitis C therapy/documentation and in week 24 of follow-up

- Metabolic syndrome

**Definition according to WHO (1999):**

*Glucose intolerance, impaired glucose tolerance (IGT) or diabetes mellitus and/or insulin resistance together with two or more of the other criteria listed below:*

Criteria:

- Impaired glucose regulation or diabetes
- Insulin resistance (highest quartiles of the HOMA-IR index)
- Dyslipidemia:

Triglycerides: ≥ 150 mg/dl (1.7 mmol/l) and/or

HDL cholesterol: ♂:< 35 mg/dl (0.9 mmol/l), ♀: < 39 mg/dl (1.0 mmol/l)

- Hypertension: ≥ 140/90 mm Hg and/or corresponding treatment
- Central obesity: waist-to-hip ratio: ♂: > 0.9, ♀: > 0.85 and/or BMI > 30 kg/m^2^

Microalbuminuria: ≥ 20 µg/min or ≥ 30 mg/g creatinine (albumin-to-creatinine ratio)

or

**Definition acc. to NCEP_III_ATP (2001) synonym ATP III:**

Criteria:

- Hypertriglyceridemia: ≥ 150 mg/dl (1.7 mmol/l)
- Lowered HDL cholesterol: ♂:< 40 mg/dl, ♀: < 50 mg/dl
- Hypertension: ≥ 130/85 mm Hg and/or corresponding treatment
- Central obesity: BMI > 30 kg/m^2^ or waist: ♂: > 102 cm, ♀: >88 cm

Elevated fasting plasma glucose: ≥ 110 mg/dl (6.1 mmol/l)

or

**Definition acc. to IDF (2005):**

*Central obesity defined as: waist: ♂: > 94 cm (European), ♀: >80 cm (European) + 2 of the following criteria*

*Central obesity defined as: waist: ♂: > 90 cm (Asian), ♀: >80 cm (Asian) + 2 of the following criteria*

*Central obesity defined as: waist: ♂: > 85 cm (Japanese), ♀: >90 cm (Japanese) + 2 of the following criteria*

*For South and Central Americans the values for Asians are used.*

*For Africans, Mediterranean inhabitants and inhabitants of the Middle East the values for Europeans are used.*

Criteria:

- Hypertriglyceridemia: ≥ 150 mg/dl (1.7 mmol/l) or corresponding treatment
- Lowered HDL cholesterol: ♂:< 40 mg/dl (0.9 mmol/l), ♀: < 50 mg/dl (1.1 mmol/l) or corresponding treatment
- Hypertension: ≥ 130/85 mm Hg or corresponding treatment
- Elevated fasting plasma glucose: ≥ 100 mg/dl (5.6 mmol/l) or type 2 diabetes
- Diabetes mellitus type ???
- Overweight
- Blood pressure parameters
- Hip and waist circumference (calculation of hip/waist ratio)
- Smoker:
  1. currently smoking: patients who have previously smoked ≥ 100 cigarettes and are currently smoking.
  2. former smokers: patients who have previously smoked ≥ 100 cigarettes and are currently not smoking.
  Nonsmokers:
  patients who have previously smoked < 100 cigarettes.
- Laboratory parameters:
  Na, K, Ca, insulin (fasting)
  (Calculation of the HOMA-IR index:
  [fasting insulin (in mIU/l) x fasting plasma glucose (in mmol/l)/ 22.5]
  or
  [fasting insulin (in mIU/l) x fasting plasma glucose (in mg/dl)/ 405]),
  (repeat presentation of HbA_1c_, HDL, LDL, triglycerides, cholesterol (total), glucose (fasting), uric acid, BMI)
- Special concomitant medication

Diabetes: insulins and insulin analogues, oral antidiabetics (e.g. alpha-glucosidase inhibitors, biguanide derivatives, sulfonylurea derivatives, "glinides", "glitazones")

Hypertension: antihypertensives (e.g. ACE inhibitors, angiotensin II antagonists, centrally acting antihypertensives, beta receptor blockers, calcium channel blockers, diuretics, combination products)

Dyslipidemia: lipid reducers (e.g. HMG-CoA reductase inhibitors, fibrates, nicotinic acid and derivatives),

Obesity: e.g. lipase inhibitors, appetite suppressants

### Additional module to record treatment of hepatitis C in patients after liver transplant

#### Baseline examination: transplant-related data

- - - - 1. *Information on donor*
- Gender, age, height, body weight, CMV (positive/negative/NA), HBV (HBsAg, anti-HBc), anti-HCV
- Donor organ quality (good, acceptable, poor); perfusion (excellent, good, acceptable, poor).
  - - - 1. *Information on recipient*
- History of primary liver disease ([selection fields: alcoholic liver disease, PBC, PSC, hepatitis B, hepatitis C, hepatitis of undetermined etiology, fulminant liver failure/toxic hepatitis, HCC, AIH, other (specify)]
- Blood group: 0, a, B, aB; Rhesus +/-
- Concomitant malignancies: HCC (yes/no)
- Specific laboratory data to calculate the MELD score before transplantation ((S-creatinine, INR, bilirubin before transplantation) 10*(0.957ln (serum creatinine)+0.378Ln (bilirubin total)+1.12ln(INR)+0.643)
- Anti-HAV (anti-HAV (IgG / IgM) negative)
- CMV status on liver transplantation (positive, negative), CMV prophylaxis/treatment [selection fields for duration]
  - - - 1. *Information on transplantation/allocation*
- Date and type of liver transplantation (LDLT, split, full-size organ); (first or retransplantation (date, cause (rejection, cirrhosis, other)), combined/other transplantation, cold ischemia time (0-30 hours)
- Initial immunosuppressive regimen in liver transplantation [selection fields: tacrolimus, cyclosporines, mycophenolate mofetil, azathioprine, sirolimus, everolimus, methylprednisolone, prednisolone, prednisone, hydrocortisone, other (specify)] including induction therapy, if any [selection fields: none, IL-2 receptor inhibitors, ATG/ATGAM/ALG, OKT3, other (specify)]
  - - 1. ***Baseline examination: Evaluation between liver transplantation and start of peg­interferon alfa-2a +/-ribavirin treatment***
- Smoking behaviour (see cardiovascular module, fade in if necessary or enter appropriately)
- Hepatitis C laboratory diagnostic tests: HCV-PCR (qualitative/quantitative), anti-HCV (last status in each case)
- CMV disease since liver transplantation up to start of peg­interferon therapy: number; viral load (PCR system), CMV syndrome, tissue invasive CMV infection; CMV prophylaxis / CMV therapy / both / no treatment
- Acute cellular rejection since liver transplantation: number, highest Banff score, highest rejection activity score, treated yes/no
- Chronic ductopenic rejection since liver transplantation:
  - ultrasound: normal findings, splenomegaly, picture of chronic liver disease, fatty liver, sonographic signs of liver cirrhosis, result not known, because performed by previous care physician
  - Histology: date of performance; stage of fibrosis and degree of inflammatory activity after selection of score
  - Fibroscan (kPa)
- Complications since liver transplantation (inquire with yes/no): bile duct stenoses (NAS/AS; ITBL), hepatic artery thrombosis, portal vein thrombosis, recurrent alcohol abuse, recurrent autoimmune mediated diseases (AIH, PBC, PSC)/ de novo AIH, HCC recurrence; HBV recurrence)
- Dialysis dependence (yes/no)
- Blood pressure parameters
- Current immunosuppressive therapy (generic names, daily dose, level)

Selection: mycophenolate mofetil, tacrolimus, cyclosporine, azathioprine, sirolimus, everolimus, prednisone, methylprednisone, prednisolone, hydrocortisone, other

For all, daily dose,

possible ranges

|  | Daily dose | Trough level |
| --- | --- | --- |
| Mycophenolate mofetil | 250 mg – 4 g | 0.5-5 mg/l |
| Tacrolimus | 5-30 mg | 3-20 ng/ml |
| Cyclosporine | 100 mg – 1 g | 30-500 ng/ml |
| Azathioprine | 50-300 mg | NA |
| Sirolimus | 3-15 mg | 3-20 ng/ml |
| Everolimus | 0.5-3 mg | 2-15 ng/ml |
| Prednisone, methyl­prednisone, prednisolone, hydro­cortisone | 1-100 mg | NA |

- Special concomitant medication:
  Erythropoietin, granulocyte stimulating factors, thrombopoietin
- Opportunistic infections in the last 52 weeks, pathogens involved: Aspergillus, Candida, Cryptococcus, herpes simplex, herpes zoster, Listeria monocytogenes, Pneumocystitis carinii, NA infection, no detectable pathogens
  - - 1. ***Examinations during hepatitis C treatment in week 12, 24, 36 and 48, at the end of hepatitis C treatment and*** ***in week 24 of follow-up***
- Dialysis dependence
- Blood pressure parameters
- Laboratory parameters
  Cholesterol, HDL, LDL, uric acid, albumin

For visit current immunosuppressive treatment (generic names, daily dose, level); selection: mycophenolate mofetil, tacrolimus, cyclosporine, azathioprine, sirolimus, everolimus, prednisone, methylprednisone, prednisolone, hydrocortisone, other

For all: daily dose

possible ranges

|  | Daily dose | Trough level |
| --- | --- | --- |
| Mycophenolate mofetil | 250 mg – 4 g | 0.5-5 mg/l |
| Tacrolimus | 5-30 mg | 3-20 ng/ml |
| Cyclosporine | 100 mg – 1 g | 30-500 ng/ml |
| Azathioprine | 50-300 mg | NA |
| Sirolimus | 3-15 mg | 3-20 ng/ml |
| Everolimus | 0.5-3 mg | 2-15 ng/ml |
| Prednisone, methylprednisone, prednisolone, hydrocortisone | 1-100 mg | NA |

- Special concomitant medication:
  Erythropoietin, granulocyte stimulating factors, thrombopoietin
- CMV infections, viral load (PCR system) and medication (prophylaxis, including duration, early therapy, therapy)
- Opportunistic infections pathogens involved: Aspergillus, Candida, Cryptococcus, herpes simplex, herpes zoster, Listeria monocytogenes, Pneumocystitis carinii, NA infection, no detectable pathogens
- Acute cellular rejection since liver transplantation: number, highest Banff score, highest rejection-activity score, treated yes/no
- Chronic ductopenic acute rejection since liver transplantation:
  - ultrasound: normal findings, splenomegaly, picture of chronic liver disease, fatty liver, sonographic signs of liver cirrhosis, result not known
  - Histology: date of performance; stage of fibrosis and degree of inflammatory activity after selection of score
  - Fibroscan (kPa)
- Loss of transplant yes/no, reasons:
- acute rejection
- chronic ductopenic rejection
- recurrence of underlying disease
- death with functioning transplant

### Pharmacoeconomics

- - - 1. ***Recording of scheduled visits***

For all visits scheduled according to the observational protocol, the following are recorded as appropriate:

- Date
- Physician contact yes/no*
- Contact with medical personnel yes/no**
- Complete blood count yes/no
- Basic blood count yes/no
- HCV-RNA determination qualitative yes/no
- HCV-RNA determination quantitative yes/no
- Determination of liver values yes/no
- Determination of thyroid values yes/no
- Determination of autoimmune antibodies yes/no
- Determination of HCV genotype yes/no
- Ultrasound
  - Normal ultrasound yes/no
  - Colour Doppler ultrasound yes/no
- Biopsy
  - Date
  - Outpatient/inpatient

Apart from the documentation of physician contact and contact with medical personnel, all data, as far as available, are taken from the main module. Additionally, the performance of an ultrasound examination (colour Doppler / normal), the determination of the thyroid values and the performance of a further liver biopsy can be reported for every visit of the main module.

*/** No differentiation is made between "visit" and telephone contact.

- - - 1. ***Recording of unscheduled visits***

For all visits held between the visits scheduled in the observational protocol, the following are recorded:

- Date period
- Physician contacts * (number)
- Contacts with medical personnel** (number)
- Complete blood count (number)
- Basic blood count (number)
- HCV-RNA determination qualitative (number)
- HCV-RNA determination quantitative (number)
- Determination of liver values (number)
- Determination of thyroid values (number)
- Ultrasound
  - Normal ultrasound (number)
  - Colour Doppler ultrasound (number)

*/** No differentiation is made between "visit" and telephone contact.

- - - 1. ***Recording of prescribed antiviral medication (hepatitis C)***
- Number of prescribed packs of Pegasys
  - Details of strength (135/180 µg)
  - Details of pack size (1/4/12 FS)
  - Details of date period of prescription
- Number of prescribed packs of Copegus
  - Details of strength (200/400 mg)
  - Details of pack size (28/42/112/168 (Copegus 200mg))
  - Details of pack size (14/56 (Copegus 400mg))
  - Details of date period of prescription
    - 1. ***Recording of prescribed concomitant medication (hepatitis C associated)***
- Number of prescribed packs of concomitant medication
  - Details of strength
  - Details of pack size
  - Details of date period of prescription
    - 1. ***Recording of prescribed HIV medication***
- Number of prescribed packs of medication for HIV therapy
  - Details of strength
  - Details of pack size
  - Details of date period of prescription
    - 1. ***Recording of prescribed substitution medication***
- Number of prescribed packs of medication for substitution
  - Details of strength
  - Details of pack size
  - Details of date period of prescription
    - 1. ***Recording of referrals to specialists***
- Date period of referral
- Number of referrals
- Choice of specialist group (selection field)
  - Dermatologist
  - Radiologists
  - Psychiatrist/neurologist
  - Endocrinologists
  - Ophthalmologist
  - Rheumatologist
  - Dentist
  - Cardiologist
  - Nephrologist
    - 1. ***Recording of cooperation with general practitioner***

The cooperation with the general practitioner is recorded in terms of

- Treatment monitoring
- Interferon prescription
- Interferon administration
- Adverse drug reaction management.
  - - 1. ***Recording of incapacity for work***
- Gainful employment yes/no
- Nature and extent of gainful employment
- Highest educational attainment
- Treatment-related sickness periods (days)

The sociodemographic data and information on treatment-related periods of sick leave are taken from the main module, their availability is assured by compulsory fields in the pharmacoeconomic module.

- - - 1. ***Recording of hospitalizations***
- Number of days in hospital associated with the HCV disease or HCV therapy
- Details of reason for admission (as free text)
- Link to the SAE reports

### Recording of adverse events and pregnancies

The following data are collected for recording adverse events:

- Description of the event
- Start and end date
- Severity (mild, moderate, severe)
- Seriousness criteria
- Outcome of the event
- Causal relationship with the treatment
- Therapy

The physician's assessment regarding whether an adverse event is serious is recorded in the case record form.

Recording of pregnancies

At least the following data are collected for recording of pregnancies (pregnancy / lactation, paternal exposure):

- Details of pregnancy and course of pregnancy
- Outcome of pregnancy (fetus)
- Seriousness criteria (fetus)
- Causal relationship with the treatment
- Information on the infant

## Influencing and interfering variables

At baseline, the year of the first diagnosis of hepatitis C, the, hepatitis C genotype, hepatitis C viral load, immune status, blood and liver values and relevant comorbidities are recorded. The clinical course parameters are evaluated taking into account their recorded baseline values. Weight, ethnicity, gender and age are documented at study outset, as these values can represent positive and negative predictive factors. In addition, any deviations in the patient distribution compared to the distribution in previous studies can be recorded and evaluated.

## Adverse events

All adverse events are documented on the sheet provided in the eCRF, regardless of whether they are serious or not, during the noninterventional study including the follow-up phase. For patients who discontinue the treatment prematurely, the adverse events are documented for up to 90 days after treatment discontinuation.

An adverse event is any untoward occurrence in an affected person administered a medication and which does not necessarily have to have a causal relationship with this treatment. These also include the following events:

- Abnormal laboratory values, insofar as they
  - are accompanied by clinical symptoms or
  - result in a change in the treatment (e.g. dose adjustment, interruption of treatment, discontinuation of treatment) or
  - require medical intervention or
  - are considered clinically relevant by the physician
- Special situations, i.e. overdose, abuse, misuse, medication error or near-misses
- Lack of efficacy (LoE) / progression of disease
- Suspected Transmission of Infectious Agent by Medicinal Product (STIAMP)
- Drug interactions
- Product quality and/or technical complaints
- Reports involving suspect counterfeit or counterfeit drugs / falsified medicinal products

## Expedited reporting procedure

According to the Roche standard, in this noninterventional study all individual cases documented as an adverse event are reported to the sponsor within 24 h (page 1 "Contact") if they fulfil the following criteria:

- serious adverse event, defined as an event which
  - is fatal or life-threatening or
  - requires hospitalization or prolongation of hospitalization or
  - results in persistent or significant disability / incapacity or
  - is a congenital anomaly / birth defect or
  - is an important medical event (*)
- (*) An important medical event is an event that may not be fatal, not life-threatening or do not require hospitalization but which may considerably impair the patient. Adverse events are also medically important if they require an intervention / treatment to prevent their occurrence which corresponds to those in the definition "serious adverse event".

The following events are reported independent of possible documentation of an adverse event in the expedited procedure:

- product quality / technical complaints
- reports involving suspect counterfeit or counterfeit drugs / falsified medicinal products
- occupational exposure (e.g. needle-stick injury in medical personnel)

Please use the enclosed bilingual AE Report Form "Adverse Event Form" for this purpose (see Annex 14.2). or alternatively the contact form at the following link:

**https://www.roche.de/service/kontakt.php**

## Pregnancies

In accordance with the Roche standard, the occurrence of pregnancies (pregnancy / lactation, paternal exposure) is recorded in the case record form during the noninterventional study and for up to 7 months after the end of the therapy phase in partners of patients and for up to 4 months in female patients after the end of the therapy phase and is additionally to be reported by the physician to Roche within 24 h using the record form provided for this purpose: "German Local Drug Safety Bilingual RO-GNE: Pregnancy Record Form (English/German)" (see Annex 14.3).

Pregnancies (pregnancy / lactation, paternal exposure) are also to be reported in the case of adverse events / adverse drug reactions. The physician is requested to advise the patient regarding the risks associated with continuing the pregnancy including the possible effects on the fetus. Pregnancies are to be followed up accordingly.

Pregnancies of the partners of affected persons are also to be documented and reported to the sponsor if an influence on the course of pregnancy cannot be ruled out because of the pharmacological behaviour of the medicinal product. Pregnant partners of affected patients should be advised in a similar manner as described above. Pregnancies are to be followed up accordingly.

# Statistical aspects

## Number of patients

Overall, it is planned to document at least 9000 patients in specialized practices and outpatient departments with experience in treating patients with interferon and ribavirin over a recruitment period of at least four years.

Since about 16,000 patients currently receive interferon therapy in outpatient departments and specialized practices in Germany every year, this results in a sample size of about 12.5 %. A representative sample can be assumed because of the chosen distribution mode within the Association of German Resident Gastroenterologists and other centres experienced in interferon treatment.

## Choice of centres

Physicians throughout the Federal Republic of German familiar with interferon therapy can participate.

## Analysis plan

The descriptive statistical evaluation is performed by the company factum GmbH.

All recorded data – depending on the type of characteristics – are presented descriptively as mean, standard deviation, minimum, maximum, median, 1^st^ and 3^rd^ quartile or their frequency distribution. Categorical characteristics are presented with absolute and relative frequencies within the individual categories.

For continuous variables multiply recorded over time, basic statistics with absolute differences between the end of observation and the baseline value are calculated.

For the tolerability analysis, adverse drug reactions are counted by frequencies using the respective preferred terms categorized according to organ systems. These survey tables are additionally stratified according to the clinical relevance of the drug effect as "serious/non-serious".

Moreover, an assessment of the clinical tolerability of the hepatitis C therapy is performed by the physician.

For the safety laboratory analysis, basic statistics over time are generated per parameter. Additionally, the frequencies of changes in categorical laboratory values (classified into "normal", "increased", "reduced") are presented per laboratory parameter between the baseline value and end of treatment and between the end of treatment and follow-up.

# Report

At the latest 12 months after the end or premature termination of the noninterventional study, a written report is generated. This report contains:

- number of patients enrolled in the study
- number of participating centres
- descriptive presentation of all recorded observational variables
- checking of the representativeness of the included centres and patients
- presentation of the effect of interfering variables and their importance for interpretation of the results
- Assessment of the results in relation to the previous recommendations for treatment
- General assessment of the study

# Administrative aspects

## Ethical advice

An expert assessment of the noninterventional study by the ethics committee is planned. The Scientific Director submits the observational protocol together with the informed patient consent document to his competent ethics committee for deliberation. Every participating physician can also receive ethical advice from his/her competent ethics committee on the basis of his/her professional obligation. The observational protocol together with the patient informed consent document can be passed on for this purpose.

The ethical consultation provided by the ethics committee is intended to ensure, among other things, that patient rights are not compromised and that the noninterventional study is designed for adding to existing knowledge.

## Legal principles and registration

The present survey is a noninterventional study pursuant to Section 4 Subsection 23 of the German Medicines Act (AMG) and is registered by Roche with the higher federal authority, the National Association of Statutory Health Insurance Physicians (KBV) and the Head Association of the Statutory Health Insurance Scheme (GKV) in accordance with Section 67 Subsection 6 AMG. The location, time and purpose of the noninterventional study is stated and the observational protocol is included. The involved physicians are identified by name to the KBV and Head Association of the GKV and a copy of the specimen agreement between Roche and the participating physicians is forwarded to the KBV and the Head Association of the GKV.

Information about the nature and amount of the compensation provided as required by Section 67 Subsection 6 AMG is forwarded, together with the report of the conclusion of the NIS, to the KBV and the Head Association of the GKV.

Roche has obtained agreement to this measure from the participating physicians on a contractual basis.

## Quality assurance measures

### Monitoring

The availability of an informed consent form signed by the patient is the precondition for the transmission of pseudonymized data. To verify that this precondition is fulfilled, authorized representatives of Roche Pharma AG may when necessary inspect this declaration of informed consent, which otherwise remains with the treating physician.

In addition, the data recorded for about 5 % of the documented patients is checked by a monitor appointed by Roche by comparing the data entered in the case record forms with the data in the patient file. The patient will be informed of this fact before participating in the noninterventional study and will be requested to give his/her consent. Only patients who have given their consent can be observed in the noninterventional study.

### Data management

The completed case record forms are checked by the commissioned institute for completeness, plausibility and adverse events. The participating physician undertakes to provide all necessary background information to his/her records on request. This is particularly important if data transfer errors are suspected.

Any questions will then be clarified by consulting the physician. The clarification of discrepancies will be documented.

The participating physician assures that the recorded details correspond to the truth.

### Archiving

The physician is responsible for the retention and archiving of all documents of the observational study for the duration of at least 10 years.

If the documentation is to be transferred to another party or to a different location for retention, Roche is to be informed of this fact by the physician in advance.

## Public study registry and publication

The noninterventional study is published in a public study registry prior to its commencement. The summary of results is to be published at the latest one year after completion (last patient / last visit) of the noninterventional study (e.g. publication and presentation at scientific congresses). This is done in consultation between the Scientific Director of the noninterventional study and Roche Pharma AG. However, this does not mean that either of the partners has the right to censor or withhold the publication.

# Reimbursement and fees

Reflecting the nature of the noninterventional study (observational study), the costs of the prescribed product and the medical services are reimbursed by the usual funding agencies as part of routine treatment. The fee for the noninterventional study is therefore to be understood exclusively as reimbursement for recording of the data. The complete documentation of the treatment data of a patient who can be documented in all modules based on the severity of his/her disease is reimbursed with 1,695.34 Euros. The fee can only be paid if the data made available are evaluable under the terms of this observational protocol.

The basis for calculation are reimbursement codes 80 and 85 of the Statutory Scale of Fees for Physicians; code 80 (EUR 17.49) is applied for a documentation effort of up to 20 minutes, whereas from a time input of 20 minutes onwards, reimbursement code 85 (EUR 29.14 for every started hour) applies. An increase factor of up to 2.3 can be applied to the codes depending on the estimated time required for the documentation.

The total amount for the complete documentation of a patient is calculated by adding up the complete documentation for the various visits in the main module hepatitis and the respective secondary modules HIV coinfection, opioid substitution, cardiovascular risk, liver transplantation and pharmacoeconomics. The assessment of the individual visits is shown in the following table.

| Module | Hepatitis | GÖA* | Factor | HIV infec-tion | GOÄ | Fac-tor | Opioid sub-stitution | GOÄ | Fac-tor | Cardio­vascular risk | GOÄ | Fac-tor | Trans­plan­ta­tion | GOÄ | Factor | Pharma­co-economics | GOÄ | Factor |
| --- | --- | --- | --- | --- | --- | --- | --- | --- | --- | --- | --- | --- | --- | --- | --- | --- | --- | --- |
| Baseline examination | 67.02 | 85 | 2.3 | 67.02 | 85 | 2.3 | 34.98 | 80 | 2 | 20.11 | 80 | 1.15 | 67.02 | 85 | 23 |  |  |  |
| Week 2 | 29.73 | 80 | 1.7 |  |  |  | 127.49 | 80 | 1 |  |  |  |  |  |  | 22.74 | 80 | 1.3 |
| Week 4 | 34.98 | 80 | 2 |  |  |  | 127.49 | 80 | 1 |  |  |  |  |  |  | 22.74 | 80 | 1.3 |
| Week 8 | 34.98 | 80 | 2 |  |  |  | 127.49 | 80 | 1 |  |  |  |  |  |  | 22.74 | 80 | 1.3 |
| Week 12 | 58.28 | 85 | 2 | 34.98 | 80 | 2 | 17.49 | 80 | 1 |  |  |  | 58.28 | 85 | 2 | 22.74 | 80 | 1.3 |
| Week 24 | 58.28 | 85 | 2 | 34.98 | 80 | 2 | 24.49 | 80 | 1.4 | 20.11 | 80 | 1.15 | 58.28 | 85 | 2 | 22.74 | 80 | 1.3 |
| Week 36 | 58.28 | 85 | 2 |  | 80 | 2 | 17.49 | 80 | 1 |  |  |  | 58.28 | 85 | 2 | 22.74 | 80 | 1.3 |
| Week 48 | 58.28 | 85 | 2 | 34.98 | 80 | 2 | 24.49 | 80 | 1.4 | 20.11 | 80 | 1.15 | 58.28 | 85 | 2 |  |  |  |
| End of treatment | 67.02 | 85 | 2.3 | 34.98 | 80 | 2 | 24.49 | 80 | 1.4 | 20.11 | 80 | 1.15 | 58.28 | 85 | 2 | 22.74 | 80 | 1.3 |
| W24 follow-up | 67.02 | 85 | 2.3 | 34.98 | 80 | 2 | 24.49 | 80 | 1.4 | 20.11 | 80 | 1.15 | 58.28 | 85 | 2 | 22.74 | 80 | 1.3 |
| **Total** | **533.88** |  |  | **241.92** |  |  | **220.37** |  |  | **100.57** |  |  | **416.70** |  |  | **181.90** |  |  |
| **Max. total per  patient** | **1695.34** |  |  |  |  |  |  |  |  |  |  |  |  |  |  |  |  |  |

* German Scale of Statutory Fees for Physicians

For incompletely documented visits that cannot be used for the evaluation under the terms of the observational protocol, relevant queries are submitted to the participating physicians through the query management that make it possible to fully present and complete the visits. If no complete documentation of a visit is achieved, the documentation is not reimbursed.

Since on the one hand prompt documentation is required and on the other hand it is usually impossible to document data electronically during a patient visit in addition to maintaining the patient file, it is advisable first to document the data in a hardcopy version of the case record form and then to transfer this version to the electronic CRF later on. Transfer to the electronic CRF can be delegated by the participating physician to a study assistant. The contracting party can contractually assign 20 % of the documentation fee to the study assistant for this purpose. 80 % of the total fee amount then remains for the contracting party. (For administrative reasons, agreement to a change in the percentage distribution between the contracting party and the study assistant is only granted in justified exceptional cases and after the agreement of the medical manager or one of his representatives.)

For the documentation of the prescribed HIV concomitant medication in the pharmacoeconomics part, a documentation effort of EUR 34.98 is reimbursed with a factor of 2 in accordance with the statutory fees for physicians.

For the documentation of the prescribed substitution medication in the pharmacoeconomics part, a documentation effort of EUR 17.49 is reimbursed with a factor of 1 in accordance with the statutory fees for physicians.

The practice characteristics can be documented once per calendar year. A documentation effort of EUR 20.11 is reimbursed for this with a factor of 1.15 in accordance with GOÄ 80. Whether this amount is reimbursed to the participating physician or study assistant is regulated in the contract concerning the performance of the NIS.

For the administration and explanation effort required for the quality of life questionnaires SF36 to be completed by the patient and the sociodemography questionnaires at 4 time points during the therapy (baseline examination, week 12, end of treatment/documentation, week 24 follow-up), a reimbursement amount of EUR 2.50 is paid per form, i.e. EU 10.00 for one complete set of questionnaires per patient. Whether this amount is reimbursed to the participating physician or the study assistant is regulated in the contract concerning the conduct of the NIS.

# Signatures

I have carefully read and reviewed this observational protocol (version 4.3 of 28-Feb-2013); I agree to the requirements and conditions it contains and agree to conduct the noninterventional study in accordance with the legal principles (German Medicines Act) and the instructions contained in the protocol.

I agree that changes to the observational protocol may only be made in the form of amendments which must be approved by Roche in writing.

|  |  | **Date** |  | **Signature** |
| --- | --- | --- | --- | --- |
| **Medical Manager/in** |  |  |  |  |
|  |  |  |  | Dr. Ulrich Alshuth,  Leader Medical Management Virology, Roche Pharma AG |
| **Study Program Manager** |  |  |  |  |
|  |  |  |  | Dr. Heike Berthold,  Study program Management, Roche Pharma AG |
| **Drug Safety** |  |  |  |  |
|  |  |  |  | Dorothea Thurn,  Drug Safety Roche Pharma AG |
| **Statistician** |  |  |  |  |
|  |  |  |  | Heike Pfeiffer-Vornkahl,  factum GmbH |
| **Scientific Director** |  |  |  |  |
|  |  |  |  | Dr. med. Dietrich Hüppe |
| **Scientific Director** |  |  |  |  |
|  |  |  |  | Dr. med. Stefan Mauss |
| **Scientific Director** |  |  |  |  |
|  |  |  |  | Dr. med. Elmar Zehnter |

# References

Summary of Product Characteristics Peginterferon alfa –2a (40KD) (Pegasys®)

Summary of Product Characteristics Ribavirin (Copegus®)

Ferenci P; Shiffman M L; Fried M W et al. (2001) Early prediction of response to 40 kDa peg­interferon alfa-2a (PEGASYS) plus ribavirin (RBV) in patients with chronic hepatitis C (CHC). Hepatology 34, 4, (Suppl.2), 351A

Fried MW, Shiffman ML, Reddy RK et al. (2002) Pegylated (40 kDa) Peginterferon alfa-2a plus Ribavirin for chronic hepatitis C virus infection. N Engl J Med 347:975-82.)

EASL International Consensus Conference on Hepatitis C (1999) Consensus Statemant. J Hepatol 30:956-61

Hadziyannis SJ, Sette HJr., Morgan TR (2004) Peginterferon-_2a and Ribavirin Combination Therapy in Chronic Hepatitis C, A Randomized Study of Treatment Duration and Ribavirin Dose. Ann Intern Med.140:346-355.

Heathcote EJ, Shiffman ML, Cooksley WG, et al. Peginterferon alfa-2a in patients with chronic hepatitis C and cirrhosis. *N Engl J Med.* 2000;343:1673-1680.

Hüppe D; Zehnter E; Alshuth U et al. (2004) Epidemiologie und Klinik von Patienten mit chronischer Hepatitis C (CHC) in Deutschland: Ergebnisse aus einer online geführten Beobachtungsstudie (AWB) in gastroenterologischen Schwerpunktpraxen. Ein Projekt im Berufsverband Niedergelassener Gastroenterologen (bng). . Z Gastro 42, 8, 917 Abstr. P318

Hüppe D; Zehnter E; Mauss S et al. (2005) Wirksamkeit von Peginterferon alfa-2a (40KD) und Ribavirin von chronischen Hepatitis C Patienten in Deutschland - ein Beitrag zur Versorgungsforschung. Z Gastro 43, 8, 853 Abstr. P263

Hüppe D; Zehnter E; Mauss S et al. (2005) Qualitätssicherung in der Behandlung der chronischen Hepatitis C (cHC): ein Abgleich der Therapie unter Alltagsbedingungen zu bestehenden Empfehlungen zur Therapie. Z Gastro 43, 8, 857-858 Abstr. P276

Hüppe D; Mauss S; John C et al. (2006) Prädiktive Faktoren bei der Behandlung von Patienten mit chronischer Hepatitis C (cHC) mit Peginterferon alfa-2a (40KD) und Ribavirin. Z Gastro 44, 1, 121 Abstr. 4.18

Hüppe D; Mauss S; John C et al. (2006) Die Viruslast als prädiktiver Faktor für die Therapie der Hepatitis C mit Peginterferon alfa-2a und Ribavirin: gilt der übliche Cut-off von 800.000 IU/ml noch? Z Gastro 44, 8, 778 Abstr. P215

Hüppe D; Mauss S; John C et al. (2006) Angstfaktor 'Therapie der Hepatitis C': Gründe, warum Patienten eine Behandlung mit Peginterferon und Ribavirin ablehnen. Z Gastro 44, 8, 797 Abstr. P275

Hüppe D; Mauss S; Boeker K et al. (2007) Zur Versorgungssituation von Patienten mit chronischer Hepatitis C (cHC) und Leberzirrhose, ein aktueller Statusbericht. Z Gastro 45, 8, 856 Abstr. P383

Meyer U; Zehnter E; Mauss S et al. (2007 Treatment of chronic hepatitis C (CHC) with peg­interferon alfa-2a (40KD) (PEG) and ribavirin (RBV) in patients older than 60 years. Gastroenterology 132, 4 (Suppl. 2), A-790 - A-791 Abstr. M1856

Neumann A U; Zeuzem S; Brunda M J; Hoffman J H (2000) Rapid viral response to treatment with pegylated (40kDa) interferon alfa-2a (Pegasys) is strongly predictive of a sustained virologic response in patients with chronic hepatitis C (CHC). Hepatology 32, 4, (Suppl.2), 318A

Torriani FJ, Rodriguez-Torres M, Rockstroh JK et al.(2004) Peginterferon Alfa-2a plus Ribavirin for Chronic Hepatitis C Virus Infection in HIV-Infected Patients. N Engl J Med 351: 438-50

Zehnter E; Mauss S; John C et al. (2005) Efficacy of peg­interferon alfa-2a (40KD) and ribavirin in patients with chronic hepatitis C in Germany - a contribution to health care research. Hepatology 42, 4 (Suppl. 1), 683A Abstr. 1233

Zehnter E; Mauss S; John C et al. (2006) Better prediction of SVR in patients with HCV genotype 1 (G1) with peg­interferon alfa-2a (PEGASYS) plus ribavirin: improving differentiation between low (LVL) and high baseline viral load (HVL). Hepatology 44, 4 (Suppl. 1), 328A Abstr. 368

Zehnter E; Mauss S; John C et al. (2006) Efficacy and safety of peg­interferon alpha-2a (40KD) (PEG) plus ribavirin (RBV) 1000-1200 mg/d in genotype 1 patients with chronic hepatitis C and 'normal' ALT levels. J Hep 44, Suppl. 2, S231 Abstr. 622

Zehnter E, Mauss S, John C et al. (2006) Efficacy and tolerability of Peg interferon alfa-2a (40 kDa) and ribavirin in GT-4 patients with chronic hepatitis C (cHC) in Germany: a contribution to health care research. Liver International 26 (Suppl. 1) 74

Zehnter E; Mauss S; John C et al. (2006) Efficacy and safety of peg­interferon alfa-2a (40KD) and ribavirin in GT-2-3 patients with chronic hepatitis C in a real world setting. Gastroenterology 130, 4 (Suppl. 2), A-841 Abstr. T1823

Zehnter E; Mauss S; John C et al. (2006) Treatment of chronic hepatitis C with peg­interferon alfa-2a (40KD) and ribavirin in patients with or without drug use. Gastroenterology 130, 4 (Suppl. 2), A-840 Abstr. T1815

Zehnter E; Mauss S; John C et al. (2006) Predictive factors in treatment of chronic hepatitis C (cHC) patients with peg­interferon alfa-2a (40KD) and ribavirin. Gastroenterology 130, 4 (Suppl. 2), A-839 Abstr. T1813

Zehnter E; Mauss S; John C et al. (2006) Efficacy and tolerability of peg­interferon alfa-2a (40KD) and ribavirin in GT-1 patients with chronic hepatitis C in Germany - a contribution to health care research. Gastroenterology 130, 4 (Suppl. 2), A-842 Abstr. T1827

Zehnter E; Mauss S; Boeker K et al. (2007) The role of glomerular filtration rate (GFR) for treatment with peg­interferon alfa 2a (PEG) and ribavirin (RBV) in patients with chronic hepatitis C (CHC). J Hep 46 (Suppl.1):656A

Zehnter E; Mauss S; Boeker K et al. (2007) Potential relevance of rapid viral response for SVR and optimisation of the treatment of hepatitis C (CHC) with peg­interferon alpha-2a (PEG) and ribavirin (RBV).J Hep 46 (Suppl. 1) S249 657A

Zehnter E; Mauss S; Boeker K et al (2007) Differences between genotype 1 patients with SVR or relapse after treatment for chronic hepatitis C (CHC) with peg­interferon alfa-2a (40KD) (PEG) and ribavirin (RBV). Gastroenterology 132, 4 (Suppl. 2), A-789 Abstr. M1850

Zeuzem S, Feinman SV, Rasenack J, et al. Peginterferon alfa-2a in patients with chronic hepatitis *C.* N Engl J Med. 2000;343:1666-1672.

Zeuzem S, Diago M, Gane E et al. (2004) Peginterferon Alfa-2a (40 Kilodaltons) and Ribavirin in Patients With Chronic Hepatitis C and Normal Aminotransferase Levels. Gastroenterology 127:1724–1732

# Annexes

## Patient information and informed consent

## German Local Drug Safety Bilingual RO-GNE: Adverse Event Form (English/German)

## German Local Drug Safety Bilingual RO-GNE: Pregnancy Record Form (English/German)
